# Supplementary material for: Chemoprevention in BRCA1 mutation carriers (CIBRAC): protocol for an open allocation crossover feasibility trial assessing mechanisms of chemoprevention with goserelin and anastrozole versus tamoxifen and acceptability of treatment
Source: BMJ Open. 2018 Dec 22;8(12):e023115. doi: 10.1136/bmjopen-2018-023115 (PMC6318512; doi:10.1136/bmjopen-2018-023115)
Supplement: Author's manuscript [file bmjopen-2018-023115.draft_revisions.pdf]

BMJ Open is committed to open peer review. As part of this commitment we make the peer review history of every article we publish publicly available.

When an article is published we post the peer reviewers' comments and the authors' responses online. We also post the versions of the paper that were used during peer review. These are the versions that the peer review comments apply to.

The versions of the paper that follow are the versions that were submitted during the peer review process. They are not the versions of record or the final published versions. They should not be cited or distributed as the published version of this manuscript.

BMJ Open is an open access journal and the full, final, typeset and author-corrected version of record of the manuscript is available on our site with no access controls, subscription charges or pay-per-view fees (http://bmjopen.bmj.com).

If you have any questions on BMJ Open's open peer review process please email info.bmjopen@bmj.com

Chemoprevention in BRCA1 mutation carriers (CIBRAC): An open allocation crossover trial assessing mechanisms of chemoprevention of Goserelin and Anastrazole versus Tamoxifen

Journal:	*BMJ Open*
Manuscript ID	bmjopen-2018-023115
Article Type:	Protocol
Date Submitted by the Author:	22-Mar-2018
Complete List of Authors:	Campbell, Aideen; Queen's University Belfast, Centre for Cancer Research and Cell Biology<br>Morris, Melanie; Belfast Health and Social Care Trust, Northern Ireland Cancer Trials Centre<br>Gallagher, Rebecca; Belfast Health and Social Care Trust, Northern Ireland Cancer Trials Centre<br>Boyd, Ruth; Belfast Health and Social Care Trust, Northern Ireland Cancer Trials Centre<br>Carson, Hazel; BRCALink NI<br>Harkin, D. Paul; Queen's University Belfast, Centre for Cancer Research and Cell Biology<br>Wielogorska, Ewa; Queen's University Belfast, Institute for Global Food Security<br>Elliott, Christopher; Queen's University Belfast, Institute for Global Food Security<br>Savage, Kienan; Queen's University Belfast, Centre for Cancer Research and Cell Biology<br>McIntosh, Stuart; Queen's University Belfast, Centre for Cancer Research and Cell Biology
Keywords:	Cancer genetics < GENETICS, PREVENTIVE MEDICINE, Breast surgery < SURGERY

SCHOLARONE™
Manuscripts

BMJ Open Submission

Title Page

Study title:

Chemoprevention in BRCA1 mutation carriers (CIBRAC): An open allocation crossover trial assessing mechanisms of chemoprevention of Goserelin and Anastrazole versus Tamoxifen

Authors:

A. M. Campbell[1], M. Morris[2], R. Gallagher[2], R. Boyd[2], H. Carson[3], D. P. Harkin[1], E. Wielogorska[4], C. Elliott[5], K. I. Savage[1], S. A. McIntosh[1]*

Affiliations:

1. Centre for Cancer Research and Cell Biology, Queen's University Belfast, Lisburn Road, Belfast, BT9 7AE.
2. Northern Ireland Cancer Trials Network, East Podium, C Floor, Belfast City Hospital, Lisburn Road, Belfast, BT9 7AB.
3. BRCALink NI, 5 Spa Grange, Ballynahinch, BT24 8PD.
4. School of Pharmacy, Queen's University Belfast, David Keir Building, 18-30 Malone Road, Belfast, BT9 5BN.
5. Institute for Global Food Security, Advanced ASSET Centre, School of Biological Sciences, Queen's University Belfast.

*Corresponding author: S. A. McIntosh (s.mcintosh@qub.ac.uk)

Keywords: BRCA1, oestrogen, chemoprevention, LHRHa, aromatase inhibitor, SERM, anastrazole, goserelin, tamoxifen.

Trial Registration: EudraCT: 2016-001087-11

Protocol version 4.0, 11/09/2017

For peer review only

For peer review only - http://bmjopen.bmj.com/site/about/guidelines.xhtml

Abstract

Introduction: BRCA1 mutation carriers have a significant lifetime risk of breast cancer, with their primary risk-reduction option being bilateral mastectomy. Preclinical work from our laboratory demonstrated that in BRCA1-deficient breast cells, oestrogen and its metabolites are capable of driving DNA damage and subsequent genomic instability, which are well-defined early events in BRCA1-related cancers. Based on this, we hypothesise that a chemopreventive approach which reduces circulating oestrogen levels may reduce DNA damage and genomic instability, thereby providing an alternative to risk-reducing surgery.

Methods and Analysis: 12 premenopausal women with pathogenic BRCA1 mutations and no previous risk-reducing surgery will be recruited from Family History Clinics. Participants will be allocated 1:1 to two arms. All will undergo baseline breast biopsies, blood and urine sampling and quality of life (QOL) questionnaires. Group A will receive goserelin 3.6mg/28 days by subcutaneous injection, plus oral anastrazole 1mg/day, for 12 weeks. Group B will receive oral tamoxifen 20mg/day for 12 weeks. Following treatment, both groups will provide repeat biopsies, blood and urine samples, and questionnaires. Following a one-month washout period, the groups will cross over; Group A receiving tamoxifen and Group B goserelin and anastrazole for a further 12 weeks. After treatment, biopsies, blood and urine samples, and questionnaires will be repeated. DNA damage will be assessed in core biopsies, whilst blood and urine samples will be used to measure oestrogen metabolite and DNA-adduct levels.

Ethics and Dissemination: This study has ethical approval from the Office of Research Ethics Committees Northern Ireland (16/NI/0055) and MHRA approval (Reference: 32485/0032/001-0001). The IMPs used in this trial are licensed and in common use, with well-documented safety information. Dissemination of results will be via high-impact journals and relevant national/international conferences. A copy of results will be offered to participants and be made available to patient support groups.

Trial Registration: EudraCT: 2016-001087-11

For peer review only - http://bmjopen.bmj.com/site/about/guidelines.xhtml

Article summary

Strengths and limitations of this study:

- Chemopreventive strategies for oestrogen-receptor negative cancers in BRCA1 mutation carriers represents a significant unmet clinical need
- Solid underpinning pre-clinical data generated within our laboratory
- Extensive patient and public involvement in study design and development
- Well-characterised base of eligible patients in Northern Ireland, known to Family History and BRCA clinics from which to recruit
- Small sample size, which means that translational endpoints are exploratory

For peer review only

For peer review only - http://bmjopen.bmj.com/site/about/guidelines.xhtml

Introduction

Women with a germline BRCA1 mutation have up to an 85% lifetime risk of developing breast cancer by age 70, with the majority of these women developing triple negative disease [1, 2]. Intriguingly, a number of studies, including a large meta-analysis, have demonstrated that risk-reducing oophorectomy significantly reduces the risk of developing breast cancer in this population by up to 50% [3-5]. Risk-reducing mastectomy is the mainstay of preventative treatment offered to BRCA1 mutation carriers, which is effective but carries significant associated costs in terms of physical and psychological morbidity and healthcare spending [6-8]. Aside from risk-reducing mastectomy, the only other risk-reduction strategy available to these women is chemoprevention.

In the United Kingdom, the National Institute for Health and Care Excellence (NICE) recommends the selective oestrogen receptor modulators (SERMs) tamoxifen and raloxifene for use in women at high-risk of developing breast cancer, on the basis that they have been demonstrated to reduce the incidence of oestrogen-receptor positive tumours [9]. However, evidence for the benefit of tamoxifen as a chemopreventive agent in BRCA1 mutation carriers is conflicting. One small study showed no benefit from tamoxifen in reducing breast cancer risk in BRCA1 mutation carriers, although it has been suggested that tamoxifen use in BRCA1 mutation carriers with breast cancer may reduce the incidence of contralateral tumours [10, 11]. In one case-control study, short term (2 year) tamoxifen use was as protective for contralateral breast cancer as a longer (5 year) course in BRCA1 and BRCA2 mutation carriers, suggesting some potential chemopreventive efficacy [12].

However, given that the majority of tumours that develop in BRCA1 mutation carriers are oestrogen receptor (ER) negative, it seems counterintuitive that SERMs will reduce breast cancer risk, given that no benefit in ER negative tumours has been shown in the chemoprevention setting with tamoxifen [9]. Clinical trial data from the IBIS-II study suggests a reduction in breast cancer risk using aromatase inhibitors in women with increased risk [13]. However, there is no direct evidence to support a benefit for this approach in BRCA1 mutation carriers.

Clearly oestrogen plays a key role in tumourigenesis in these patients, as tumours develop preferentially in the oestrogen-rich tissues of the breast and ovary.

Preclinical data from our laboratory demonstrates that oestrogen and oestrogen metabolites cause DNA double strand breaks (DSBs) in ER negative breast cells (normal and cancer cell lines as well as primary breast progenitor cells isolated from patients), and that BRCA1 is necessary for repression and repair of these DSBs [14]. The implication of these findings is that in BRCA1-deficient breast cells, exposure to oestrogen and its metabolites is capable of driving genomic instability, a well-defined early event in BRCA1-related cancer development. Taken with evidence from clinical trials, which demonstrate no benefit from SERMs in reducing the incidence of ER negative tumours, this suggests that SERMs are unlikely to be effective chemopreventive agents in BRCA1 mutation carriers (who are more likely to develop ER negative tumours). Our data suggests that a chemopreventive approach which lowers circulating oestrogen levels and reduces exposure to oestrogen metabolites within breast tissue may be more successful by removing this driver of genomic instability. In post-menopausal women (or pre-menopausal women who have undergone oophorectomy), the use of an aromatase inhibitor may be considered. In premenopausal women with intact ovaries, the induction of reversible ovarian suppression using a luteinising hormone releasing hormone agonist (LHRHa) in combination with an aromatase inhibitor (AI), to suppress subsequent aromatase-mediated oestrogen production, may be effective.

Progesterone signaling has also recently been implicated in BRCA1-related tumourigenesis, with the TNF-superfamily member RANK and its corresponding ligand RANKL functioning as key factors in this pathway and potential targets for chemoprevention [15]. Importantly, LHRHa suppression of the hypothalamic-pituitary axis will also result in decreased progesterone levels, which will consequently reduce progesterone-dependent RANK/RANKL signaling. Additionally, expression of the progesterone receptor (PR), a critical mediator of the progesterone/RANK/RANKL pathway, is itself driven by

For peer review only - http://bmjopen.bmj.com/site/about/guidelines.xhtml

oestrogen signaling through the ER. Taken together, the suppression of oestrogen is likely to provide chemopreventive potential, similar to or surpassing that demonstrated through RANK/RANKL suppression.

Although LHRHa, AIs and SERMs are used in breast cancer and fertility treatments, there is no published data in the context of BRCA1-specific chemoprevention where the intention is to prevent ER negative breast cancer.

It is known that compliance and acceptability of these agents in the adjuvant setting of breast cancer treatment is good, albeit with a high frequency of side effects [16].

The compliance of women taking SERMs for chemoprevention is sub-optimal, with approximately 40% of women unable to complete 5 years of therapy because of side effects [17]. Therefore, compliance with LHRHa in combination with an aromatase inhibitor in the chemopreventive setting must be assessed and compared with SERM data as an important consideration in the feasibility of use in this context. In line with this, this study aims to inform future tolerability and compliance evaluation in a cohort who may require long durations of treatment and have a long life expectancy.

If the results from this study demonstrate that these treatments are a feasible and tolerable strategy, and that DNA damage is reduced through reduced levels of circulating oestrogen, this would provide data to support the development of a phase III study evaluating the use of this chemopreventive strategy in BRCA1 mutation carriers.

For peer review only

For peer review only - http://bmjopen.bmj.com/site/about/guidelines.xhtml

Methods/Analysis

The aims, design and setting of the study

The principal aim of this research is to establish if these treatments are tolerable, and to evaluate patient compliance. Secondary aims include tolerability of treatments and quality of life.

As exploratory objectives, this research seeks to not only confirm the link between oestrogen exposure and DSBs and generate further insight into how oestrogen and its metabolites affect tumourigenic transformation, but also to generate the preliminary data necessary to warrant a larger clinical study to evaluate chemoprevention in BRCA1 mutation carriers.

Study aims and objectives are summarised in Table 1.

Design

A schematic overview of the trial is depicted in Figure 1.

The crossover design allows comparison of outcomes from both treatments to the participant baseline, thus each participant acts as their own control for both treatment arms.

Blinding or masking is not achievable given the nature of administration of goserelin (subcutaneous implant).

Given that each participant will receive both treatments without blinding, randomization was felt unnecessary and alternate allocation of treatment groups in order of recruitment is adequate.

The setting of the trial in tertiary care (Family History Breast Clinics) reflects the main location for the ongoing care and management of this cohort.

Selection of participants

Participants will be recruited from Family History Breast Clinics within Northern Ireland as identified by clinicians and by posters and publicity regarding the trial presented and distributed at relevant public engagement events.

The participant inclusion and exclusion criteria are outlined in Table 2. The Chief Investigator (or appropriately trained delegate) will confirm eligibility against the inclusion/exclusion criteria.

Description of Interventions

Goserelin and Anastrazole Treatment

Participants will receive goserelin 3.6mg by subcutaneous injection every 28 days for 12 weeks, simultaneously receiving oral anastrazole 1mg per day for 12 weeks.

Tamoxifen Treatment

Participants will receive oral tamoxifen 1mg per day for 12 weeks.

Washout Period

Upon completion of the first treatment arm, participants will have a 4-week period of no treatment to allow washout of active substances, before commencing second treatment arm.

Description of Processes

Procedure for obtaining informed consent and trial arm allocation

Participants will receive an adequate explanation of the objectives, methods, potential risks and anticipated benefits of the study. They will be given a current, ethically approved participant information sheet (PIS) for their consideration. Following this, informed consent will be obtained once women have had a minimum of 24 hours to consider trial entry and the opportunity to ask further questions. Following consent, participants will be provided with a copy of the signed consent

For peer review only - http://bmjopen.bmj.com/site/about/guidelines.xhtml

form, with a copy filed in the patient notes, and the original retained in the site file, available for inspection if required.
No protocol required assessments will be conducted until the consent form has been signed and dated by both participant and investigator.
Participants will be provided the option to allow the use of blood samples, other body fluids, and tissues obtained during testing, operative procedures, or other standard medical practices for further research purposes.
Participants will be alternately allocated to treatment arms.

Replacement of participants
In the case of a participant request for withdrawal or failure to collect biological samples, that participant will be withdrawn from the study and a new participant enrolled into the treatment arm the original participant has been retracted from.

Percentage Consenting to Trial Entry
Records will be kept of all the Patient Information Sheets distributed to allow calculation of percentage of patients consenting to trial entry.

Compliance Measurement
Participants will receive a medication diary card for each oral treatment at the same time the tablets (Anastrazole for Arm A and Tamoxifen for Arm B) are issued to track compliance.

Concomitant medication:
Participants must be instructed not to take any medications (including herbal and over-the-counter remedies) during the study without prior consultation with the investigator.
Any hormonal treatments (including all forms of hormonal contraception) are prohibited.
Concomitant medications must be recorded in the patients' notes at the time of initial consultation and any amendments documented at subsequent visits.

Quality of Life Assessment
Questionnaires will be completed on Day 1 (baseline), Day 84 (+/-3days, following the end of first arm) and Day 196 (+/-3days following the end of second arm).
Two questionnaires will be used to assess health-related Quality of Life (QOL):
1. EORTC QLQ-C30 questionnaire assessing quality of life
2. EORTC QLQ-BR23 questionnaire assessing treatment side effects and sexual dysfunction

Recording of Adverse Events
An Adverse Event (AE) is any untoward medical occurrence (including deterioration of a pre-existing medical condition) in a subject administered an Investigational Medicinal Product (IMP). The event does not necessarily have a causal relationship with treatment or usage.
An Adverse Reaction (AR) is an AE that is suspected as having a causal relationship to the IMP.
Each AE and its causality, severity, and expectedness are assessed by the chief investigator (or nominated representative) and recorded in the patient notes.
All IMPs in this study are licensed with well-defined safety profiles. As such, we will only record data relating to ARs in the case report form (CRF).
If the event is classified as serious (SAE) the reporting procedures as per Belfast Health and Social Care Trust will also be followed.
Follow-up will continue until all the necessary safety data for the event has been gathered and until the AR or SAE has resolved, returned to baseline or stabilised.

For peer review only - http://bmjopen.bmj.com/site/about/guidelines.xhtml

Pregnancy Testing

Participants will be asked to provide a non-sterile urine sample (the initial stream) in a clean, non-sterile receiver. The sample will be used to perform a urinary HCG test in the clinic following manufacturer's SOP.

Pregnancy testing will be performed twice:

1. At the time of assessment of eligibility for the trial following consent, prior to the first arm treatment commencement
2. On Day 112 +/-3 (week 16), prior to starting the second treatment arm

Core Biopsies

Biopsy samples will be collected as standard using a 14G core biopsy needle under ultrasound guidance in the Department of Radiology at Belfast City Hospital. Two core biopsies will be sharply dissected into at least 4 smaller samples (approx. 5mm^3 each) and placed in individual Eppendorf tubes, then snap frozen with liquid nitrogen and stored for later use. Two core biopsies will be placed in individual formalin-containing sample pots, after which they will be Formalin Fixed Paraffin Embedded (FFPE) as per standard operating procedures within the Belfast Trust.

Core biopsies will be taken on Day 1 (baseline), Day 84 (+/-3days following end of first arm) and Day 196 (+/-3days following end of second arm).

Histopathological Analysis

One FFPE sample will be assessed by a histopathologist to ensure adequate sampling of glandular tissue.

Comet Assays

Two frozen biopsy specimens will be used to perform tissue-based comet assays to assess DNA damage.

Immunohistochemistry

One FFPE core biopsy sample will be sectioned and stained with antibodies against 53BP1 and γH2AX using conventional and fluorescent immunohistochemistry to assess DNA DSBs in these samples.

Additional sections will also be stained for RANK and RANKL as well as Ki67 using immunohistochemistry.

Mass Spectrometry

Two frozen biopsy specimens will be used to examine concentrations of oestrogen metabolites 2-hydroxyestradiol (2-OHE$_2$) and 4-hydroxyestradiol (4-OHE$_2$), using UPLC-MS/MS.

Serum Samples

Serum sampling will be performed on Day 1 (baseline), Day 84 (+/-3 days following end of first arm) and Day 196 (+/-3 days following, end of second arm). Participants will undergo venepuncture with blood samples collected in two silica (Clot Activator) coated 10ml blood sample tubes. After the minimum recommended clotting time (60 minutes), blood samples will be centrifuged to allow separation of serum from blood cells, with the serum stored at -80°C in 1ml aliquots in RNAse free tubes. Following sample accrual, samples will be thawed and serum 2-OHE$_2$ and 4-OHE$_2$ levels and levels of depurinating DNA adducts (common types of DNA damage caused by oestrogen metabolites) will be measured using UPLC-MS/MS.

Urine Samples

Urine sampling will be performed on Day 1 (baseline), Day 84 (+/-3 days following, end of first arm) and Day 196 (+/-3 days following, end of second arm). Mid-stream urine samples will be collected in

For peer review only - http://bmjopen.bmj.com/site/about/guidelines.xhtml

sterile containers. Within 2 hours of collection samples will be centrifuged to separate debris from the urine and then stored at -80°C in 500µl aliquots in RNAse free tubes. Following sample accrual. Samples will be thawed and urinary 2-OHE_2 and 4-OHE_2 levels and levels of depurinating DNA adducts will be measured using UPLC-MS/MS.

Whole Blood Sampling

Each participant will provide one whole blood sample at one time point in the study. This will be performed at baseline (Day 1), unless the patient is already enrolled and receiving treatment, in which case the whole blood sampling will be performed at the end of the treatment arm that they are currently receiving (Day 84 +/-3d or Day 196 +/-3d).

The whole blood samples will be collected at the same venepuncture session as serum sampling. Blood will be collected into one EDTA coated tube and frozen/stored at -80^{0}C in 1ml aliquots. Samples will be retained for future use including DNA extraction.

Timeframe for interventions and assessments is summarized in Table 3.

Comparisons

Each participant acts as her own control. Results from each individual's set of samples (core biopsies, serum and urine) and questionnaires, taken at baseline end of first arm and end of second arm will be compared. Results will not be compared between patients.

Statistical Analysis

This study is primarily aimed to determine acceptability and tolerability of the proposed chemopreventive treatments. Therefore, no formal sample size calculation has been carried out, as the laboratory end-points are all exploratory.

For these reasons, no formal statistical analysis is planned. Nonetheless, descriptive statistics will be used to summarise the principal and experimental findings of the study.

Laboratory analysis:

The proposed translational studies include performing comet assays on fresh frozen breast core biopsies at baseline and after each treatment arm. The comet assay protocol has been optimized on breast cell cultures as well as fresh frozen breast biopsy samples using neutral lysis techniques with automated scoring performed on a high content screening system. FFPE breast core biopsy blocks are created at the same time-points as fresh-frozen samples. Formation of γ-H2AX in response to DNA double stranded breaks (DSBs) provides the basis for a sensitive immunohistochemical assay of DNA damage in human biopsies and has been optimised in FFPE breast samples with simultaneous staining for 53BP1, which localises to DNA strand breaks, with foci numbers proportional to DNA damage levels. Based on our hypothesis, the anticipated decrease in measurable DNA damage in response to oestrogen suppression would support the use of this strategy as a chemoprevention option in BRCA1 mutation carriers.

Further FFPE sections will be analysed for Ki67, RANK and RANKL expression to assess the effect of treatment on these markers of proliferation (Ki67) and progesterone signalling (RANK/RANKL). As the LHRHa used to suppress oestrogen will also result in progesterone suppression, it is expected that RANK and RANKL expression will also decrease. Given that inhibition of RANK has been shown to reduce breast cell proliferation in BRCA1 mutation carriers, it is possible that LHRHa treatment might be an alternative way of down regulating this pathway and potentially decreasing the development of highly proliferative cells with grossly aberrant DNA repair observed in BRCA1 precancerous tissues with high levels of RANK/RANKL expression [15].

For peer review only - http://bmjopen.bmj.com/site/about/guidelines.xhtml

Oestrogen metabolite levels in serum and urine will be measured using ultra performance liquid chromatography tandem mass spectrometry (UPLC-MS/MS) and have been optimised on healthy volunteer samples with permission from Queen's University Ethics Committee.

Patient and public involvement:

BRCA1 and BRCA2 mutation carriers were involved throughout the design process via the Northern Ireland BRCA mutation carrier support group, BRCALink NI. The study design was finalized with input from a BRCA patient advocate focus group, where the consensus was that inclusion of an active control treatment was more acceptable than placebo. Women within the group had experienced tamoxifen therapy as part of cancer treatment and deemed potential side effects acceptable in the context of risk reduction. The BRCA mutation carrier cohort in Northern Ireland is overwhelmingly enthusiastic about chemoprevention clinical trials, resulting in a highly motivated patient group keen to contribute to future solutions to BRCA-related breast cancer risk. In keeping with this, the trial management group (TMG) for this study includes a BRCA1 mutation carrier.

Data Collection and Management:

The Investigator and study site staff will ensure that data collected on each subject is recorded on the Case Report Form (CRF) as accurately and completely as possible. Data management processes will be fully defined within the Data Management Plan for the study, held within the trial master file (TMF).

Trial Management

Trial Management Group (TMG)
The TMG will consist of an independent chair, the Chief Investigator, Co-investigators and clinical research nurse as well as other relevant committee members as defined within the TMG charter. The TMG will provide overall supervision of the trial on behalf of the trial funder and sponsors, and ensure that the trial is conducted according to Good Clinical Practice guidelines. The TMG will focus on progress of the trial, adherence to trial protocol, and patient safety. The TMG will report to the BHSCT CTIMP Oversight Committee.
Meetings frequency will be defined within the TMG Charter but as a minimum will meet at least quarterly.

Trial Steering Committee (TSC)
The Belfast Health and Social Care Trust CTIMP Oversight Committee, which meets quarterly, acts as the TSC for CIBRAC.

Independent Data Monitoring Committee (IDMC)
After review by the Sponsor, it was not considered that an IDMC was required for this trial due to its low risk nature, in that the IMPs are not novel agents, and are licensed agents with a well-defined safety profile.

Monitoring
The Sponsor has appointed the Clinical Research Monitoring Service within the Northern Ireland Clinical Trials Unit to undertake on site monitoring. An initial monitoring visit was carried out prior to the release of the final permissions letter by the Sponsor. The first interim monitoring visit will take place within 6 months of the first patient being recruited. Further monitoring visits will take place approximately every 6 months from the date of the first visit. A close-out visit will occur once the last patient visit has been completed. A trial monitoring plan is available within the TMF.

For peer review only - http://bmjopen.bmj.com/site/about/guidelines.xhtml

The SPIRIT checklist has been used in writing this report[18].

For peer review only

For peer review only - http://bmjopen.bmj.com/site/about/guidelines.xhtml

Ethics and dissemination

Ethics: The Health and Social Care Research Ethics Committee B (HSC REC B), as part of the Office for Research Ethics Committees Northern Ireland (ORECNI), awarded ethical approval for this study (REC Reference:16/NI/0055, 21/4/16). A summary of amendments is given in Table 4.

The Investigational Medical Products used in this study are all licensed agents, which are in common use in the treatment of breast cancer. They therefore have well-defined safety profiles available.
The women offered the opportunity to participate in this study are not cancer patients, but rather are healthy pre-menopausal women who carry a pathogenic mutation in the BRCA1 gene. Although this is effectively a healthy volunteer study, it was felt it would not be appropriate to offer incentives for participation, although travelling expenses are covered for trial hospital visits.
There was extensive patient and public involvement in the study design and development, to ensure that the proposed treatments and study procedures would be acceptable to patients.

Dissemination: Academic dissemination of the study will be via publication in peer-reviewed journals in the relevant field (the ICMJE criteria for authorship will be followed), and by presentation at appropriate national and international meetings. These may include meetings such as the biannual Symposium on Hereditary Breast and Ovarian Cancer in Montreal, Canada. A lay summary of the study results will be available, and all participants will be offered access to a copy of this. Furthermore, this will be shared with our partner PPI organization BRCALink NI and disseminated through their website and social media channels. All mass spectrometry and sequencing data generated from this study and/or the future use of study samples will be deposited in appropriate publically-accessible repositories, with the associated anonymised meta-data.

Word count: 3536 words

For peer review only

For peer review only - http://bmjopen.bmj.com/site/about/guidelines.xhtml

List of Abbreviations

AE	Adverse Event
AI	Aromatase Inhibitor
AR	Adverse Reaction
ASNTT	Aseptic Non-touch Technique
BRCA1	Breast Cancer 1 Early Onset Gene
BRCA2	Breast Cancer 2 Early Onset Gene
BRCALink NI	Northern Ireland Support Group for BRCA1 & BRCA2 mutation carriers
CCRCB	Centre for Cancer Research and Cell Biology
CI	Chief Investigator
CIBRAC	Chemoprevention in BRCA1 mutation Carriers Study
CRF	Case Report Forms
DNA	Deoxyribonucleic Acid
DSB	Double Strand Break
EDTA	Ethylenediaminetetraacetic acid
EORTC QLQ-C30	European Organisation for Research and Treatment of Cancer Questionnaire to Quality of Life of Cancer patients
EORTC QLQ-BR23	European Organisation for Research and Treatment of Cancer Questionnaire module to assess quality of life of breast cancer patients and supplement EORTC QLQ-C30
ER	Oestrogen Receptor
FFPE	Formalin-fixed paraffin-embedded
HSC REC B	Health and Social Care Research Ethics Committee B
IBIS-II	International Breast Cancer Interventional Study - Prevention
IMP	Investigational Medicinal Product
Ki67	Antigen KI-67, marker of cellular proliferation
LHRHa	Lutenizing Hormone Releasing Hormone Agonist
NICE	National Institute for Clinical Excellence
ORECNI	Office for Research Ethics Committees Northern Ireland
Participant	An individual who has given written informed consent and is participating in trial related activities
Patient	A patient under care who may be eligible for the trial but has not yet consented to participate in any trial related activities
PIS	Patient Information Sheet
PR	Progesterone Receptor
QOL	Quality of Life
RANK	Receptor Activator of Nuclear Factor κ B
RANKL	Ligand of Receptor Activator of Nuclear Factor κ B
REC	Research Ethics Committee
RNAse	Ribonuclease
SAE	Serious Adverse Event
SAR	Serious Adverse Reaction
SERM	Selective Oestrogen Receptor Modulator
SOP	Standard Operating Procedure

For peer review only - http://bmjopen.bmj.com/site/about/guidelines.xhtml

TMG	Trial Management Group
TNF	Tumour Necrosis Factor
UPLC-MS/MS	Ultra Performance Liquid Chromatography Tandem Mass Spectrometry
2-OHE_2	2-hydroxyestradiol
4-OHE_2	4-hydroxyestradiol
53BP1	P53 Binding Protein 1
YH2AX	Phosphorylated Histone A2 Variant X

For peer review only

For peer review only - http://bmjopen.bmj.com/site/about/guidelines.xhtml

Declarations

Author's contributions
SMcI and KS generated the study idea and hypothesis. AC was responsible for writing the protocol, supervised by SMcI and KS.
MM, RG, RB and HC contributed to protocol development, writing and revision.
EW, CE and DPH contributed to experimental design and validation. DPH also contributed to protocol development.
SMcI is the Chief Investigator of the study and has overall responsibility for its conduct.
All authors have seen and approved the final manuscript.

Funding statement
This work is supported by Cancer Research UK as part of a Clinical Fellowship award. The funding body have no input into the design of the study, the collection, analysis or interpretation of data, nor into the writing of the manuscript.

Competing interests
The authors declare they have no competing interests.

Acknowledgements
We are grateful for the contribution of our patient advocates in developing the study, particularly Hazel Carson of BRCALink NI.

Trial Sponsor
Belfast Health and Social Care Trust (BHSCT)
Sponsorship Contact Details:
Alison Murphy,
Belfast Health and Social Care Trust
Research & Development Office, Room 2010, 2nd Floor,
King Edward Building,
Royal Hospitals Site,
Grosvenor Road,
Belfast,
BT12 6BA.

Role of sponsor: The study sponsor undertook peer review of the protocol. However, the sponsor had no role in study design, nor will they be involved in data collection, analysis, interpretation of data, report writing or decisions in respect of publication.

For peer review only - http://bmjopen.bmj.com/site/about/guidelines.xhtml

References

1. Antoniou, A., et al., *Average risks of breast and ovarian cancer associated with BRCA1 or BRCA2 mutations detected in case Series unselected for family history: a combined analysis of 22 studies.* Am J Hum Genet, 2003. **72**(5): p. 1117-30.
2. Lakhani, S.R., et al., *Prediction of BRCA1 status in patients with breast cancer using estrogen receptor and basal phenotype.* Clin Cancer Res, 2005. **11**(14): p. 5175-80.
3. Heemskerk-Gerritsen, B.A., et al., *Breast Cancer Risk After Salpingo-Oophorectomy in Healthy BRCA1/2 Mutation Carriers: Revisiting the Evidence for Risk Reduction.* J Natl Cancer Inst, 2015. **107**(5).
4. Eisen, A., et al., *Breast cancer risk following bilateral oophorectomy in BRCA1 and BRCA2 mutation carriers: an international case-control study.* J Clin Oncol, 2005. **23**(30): p. 7491-6.
5. Rebbeck, T.R., N.D. Kauff, and S.M. Domchek, *Meta-analysis of risk reduction estimates associated with risk-reducing salpingo-oophorectomy in BRCA1 or BRCA2 mutation carriers.* J Natl Cancer Inst, 2009. **101**(2): p. 80-7.
6. Ludwig, K.K., et al., *Risk reduction and survival benefit of prophylactic surgery in BRCA mutation carriers, a systematic review.* Am J Surg, 2016. **212**(4): p. 660-669.
7. Hallowell, N., et al., *Looking different, feeling different: women's reactions to risk-reducing breast and ovarian surgery.* Familial Cancer, 2012. **11**(2): p. 215-224.
8. Robertson, S.A., et al., *Resource implications of risk-reducing mastectomy and reconstruction.* (1532-2157 (Electronic)).
9. Cuzick, J., et al., *Selective oestrogen receptor modulators in prevention of breast cancer: an updated meta-analysis of individual participant data.* The Lancet, 2013. **381**(9880): p. 1827-1834.
10. King, M.C., et al., *Tamoxifen and breast cancer incidence among women with inherited mutations in BRCA1 and BRCA2: National Surgical Adjuvant Breast and Bowel Project (NSABP-P1) Breast Cancer Prevention Trial.* Jama, 2001. **286**(18): p. 2251-6.
11. Gronwald, J., et al., *Tamoxifen and contralateral breast cancer in BRCA1 and BRCA2 carriers: an update.* Int J Cancer, 2006. **118**(9): p. 2281-4.
12. Gronwald, J., et al., *Duration of tamoxifen use and the risk of contralateral breast cancer in BRCA1 and BRCA2 mutation carriers.* Breast Cancer Res Treat, 2014. **146**(2): p. 421-7.
13. Cuzick, J., et al., *Anastrozole for prevention of breast cancer in high-risk postmenopausal women (IBIS-II): an international, double-blind, randomised placebo-controlled trial.* Lancet, 2014. **383**(9922): p. 1041-8.
14. Savage, K.I., et al., *BRCA1 deficiency exacerbates estrogen-induced DNA damage and genomic instability.* Cancer Res, 2014. **74**(10): p. 2773-84.
15. Nolan, E., et al., *RANK ligand as a potential target for breast cancer prevention in BRCA1-mutation carriers.* Nat Med, 2016. **22**(8): p. 933-9.
16. Bernhard, J., et al., *Patient-reported outcomes with adjuvant exemestane versus tamoxifen in premenopausal women with early breast cancer undergoing ovarian suppression (TEXT and SOFT): a combined analysis of two phase 3 randomised trials.* The Lancet Oncology, 2015. **16**(7): p. 848-858.
17. Roetzheim, R.G., et al., *Acceptance and adherence to chemoprevention among women at increased risk of breast cancer.* Breast, 2015. **24**(1): p. 51-6.
18. Chan, A.W., et al., *SPIRIT 2013 statement: defining standard protocol items for clinical trials.* Ann Intern Med, 2013. **158**(3): p. 200-7.

For peer review only - http://bmjopen.bmj.com/site/about/guidelines.xhtml

Figure 1: CIBRAC trial schema

For peer review only

For peer review only - http://bmjopen.bmj.com/site/about/guidelines.xhtml

Table 1: Trial objectives and endpoints

**Primary Objective**	**Primary Endpoint**
To establish the acceptability of trial treatments as a chemopreventive strategy in BRCA1 mutation carriers and compliance.	Percentage of patients given Patient Information Sheet (PIS) who consent to trial entry.<br>Measurement of compliance with treatment using patient medication cards.
**Secondary Objective**	**Secondary Endpoint**
To establish tolerability of trial treatments and procedures	Measurement of patient Quality of Life using questionnaires at baseline and after completion of each treatment arm.<br>Recording of Adverse Events (AEs) that occur during treatment
**Exploratory Objectives**	**Exploratory Endpoints**
To establish the potential of chemopreventive agents to reduce oestrogen mediated DNA damage in breast tissue in BRCA1 mutation carriers	Measurement of DNA damage in breast tissue using comet assays and immunohistochemical assays at baseline and after completion of each treatment arm.
To establish effect of treatment on oestrogen metabolite levels in breast tissue, urine and serum.	Measurement of breast, blood and urinary oestrogen metabolite levels using UPLC-MS/MS at baseline and after completion of each treatment arm.
To explore the mechanism of oestrogen mediated DNA damage in breast tissue in BRCA1 mutation carriers.	Analysis of breast tissue samples using alternative methods of DNA damage assessment.
To explore the relationship between oestrogen metabolite concentration in serum and urine and DNA damage in breast tissue in BRCA1 mutation carriers.	Analysis of breast tissue, urine and blood samples for proxies of oestrogen levels and DNA damage.
To explore potential biomarkers of chemoprevention efficacy.	Analysis of breast tissue, urine and blood samples for potential biomarkers of chemopreventive efficacy.

For peer review only

For peer review only - http://bmjopen.bmj.com/site/about/guidelines.xhtml

Table 2: Inclusion and exclusion criteria

Inclusion Criteria	Exclusion Criteria
Age >18 years	BRCA1 mutation of uncertain significance
Premenopausal	Personal history of breast or ovarian carcinoma
Known pathogenic BRCA1 mutation	Previous risk-reducing breast or ovarian surgery
Intact ovaries	Post-menopausal status
No previous breast/ovarian carcinoma	Concomitant use of alternative chemoprevention regimens
No other previous malignancy	Concomitant use of other hormonal agents less than one month prior to enrolment
No previous use of chemoprevention	Contraindications to study drug therapies
Willingness to use non-hormonal contraception	Contraindications to breast core biopsies
	Pregnancy or breastfeeding
	Inability to give informed consent
	Having made a decision to proceed with risk reducing surgery

For peer review only

For peer review only - http://bmjopen.bmj.com/site/about/guidelines.xhtml

Table 3: Summary of assessments and interventions

Time Point	<30 days prior to entry	Week 1	Week 4	Week 8	Week 12	Week 16	Week 20	Week 24	Week 28	Week 32 (EOT)
**Enrolment**										
Informed Consent	A+B									
Baseline History	A+B									
Eligibility Screen & Pregnancy Test		A+B				A+B				
Treatment Allocation		A+B								
Conmeds	A+B	A+B	A	A	A+B	A+B	B	B	A+B	A+B
**Interventions**										
Start Tamoxifen (12 weeks)		B				A				
Start Anastrazole (12 weeks)		A				B				
Goserelin Implant Injection		A	A	A		B	B	B		
**Assessments**										
QOL Assessment		A+B			A+B				A+B	
Core Breast Biopsies x3		A+B			A+B				A+B	
Blood samples for oestrogen & metabolite levels		A+B			A+B				A+B	
Blood sample for whole blood		A+B			A+B				A+B	
Urine samples for oestrogen & metabolite levels		A+B			A+B				A+B	
AE assessment	A+B	A+B			A+B	A+B			A+B	A+B

For peer review only

Table 4: Amendment chronology

Amendment Number	Summary of Amendment	Documents Impacted (and version)	Date sent for classification	Date Classified	Date sent to MHRA	Date of MHRA approval	Date sent to Ethics	Date of Ethics Approval	Date sent to R and D	Date of R and D approval	Effective Date
**1**	contraceptive advice amended	Protocol 1.2 dated 24-May-16	04-Sep-16	Minor 16-Sept-16	N/A	N/A	21-Jun-16	21-Jun-16	04-Sep-16	N/A	N/A
**2**	changes in the priority of existing objectives & endpoints	Protocol2.0 dated 30-Aug-16 PIS ICF 1.1 dated 19-Apr-16	04-Sep-16	Substantial 16-Sept-16	N/A	N/A	23-Sep-16	27-Sep-16	11-Oct-16	19-Jan-17	N/A
**3**	type of blood amended and volume	Protocol 2.1 dated 09-Jan-17	09-Jan-17	Substantial 30-Jan-2017	N/A	N/A	07-Feb-17	01-Mar-17	01-Mar-17	14-Mar-17	14-Mar-17
**Minor 20-Jan-17**	addition of Keith Lowry and Lesley McFaul	Not applicable	N/A	N/A	N/A	N/A	N/A	N/A	20-Jan-17	13-Feb-17	13-Feb-17
**4**	Sample Collection change	Protocol 3.0	04-Apr-17	Substantial 08/05/2017	N/A	N/A	08-May-17	02-Jun-17	10-May-17	14-Jun-17	15-Jun-17
**5**	Letter to potential participants, modify exclusion criteria	Protocol 4.0 dated 11/9/17									
**Letter 1.0 dated 11/09/17**	11-Sept-17	Substantial 21/09/17	N/A	N/A	29/09/17	10-Oct-2017	10-Oct-2017	09-Nov-2017	16-Nov-2017		

For peer review only - http://bmjopen.bmj.com/site/about/guidelines.xhtml

Trial registration data:

Data category	Information
Primary registry and trial identification number	EudraCT: 2016-001087-11
Date of registration in primary registry	9th March 2016
Sources of monetary or material support	Supported by a Cancer Research UK Clinical Research Fellowship
Sponsor	Belfast Health & Social Care Trust
Contact for public queries	AMC (a.campbell@qub.ac.uk)
Contact for scientific queries	SMcI, KS (s.mcintosh@qub.ac.uk, k.savage@qub.ac.uk)
Public title	CIBRAC: Chemoprevention in BRCA1 mutation carriers
Scientific title	Chemoprevention in BRCA1 mutation carriers (CIBRAC): An open allocation crossover trial assessing mechanisms of chemoprevention of Goserelin and Anastrazole versus Tamoxifen
Countries of recruitment	United Kingdom (Northern Ireland)
Interventions	Tamoxifen 20mg daily (oral)<br>Goserelin 3.6mg every 28 days (sc) plus anastrazole 1mg daily (oral)
Key inclusion criteria	Ages eligible for study ≥18 years<br>Sexes eligible for study: female<br>Known pathogenic germline BRCA1 mutation<br>Pre-menopausal<br>No previous breast or ovarian risk-reducing surgery
Key exclusion criteria	Personal history of breast or ovarian carcinoma<br>Previous risk-reducing breast or ovarian surgery<br>Post-menopausal status
Study type	Interventional<br>Allocation: alternate treatment allocation, non-randomised, non-blinded<br>Primary purpose: prevention<br>Phase II
Date of first enrolment	May 2017
Recruitment status	Recruiting
Primary outcome	Establish the acceptability of trial treatments as a chemopreventive strategy in BRCA1 mutation carriers, as measured by number of patients entering trial and compliance with treatment
Key secondary outcomes	To establish tolerability of trial treatments and procedures

For peer review only - http://bmjopen.bmj.com/site/about/guidelines.xhtml

Figure 1: CIBRAC trial schema

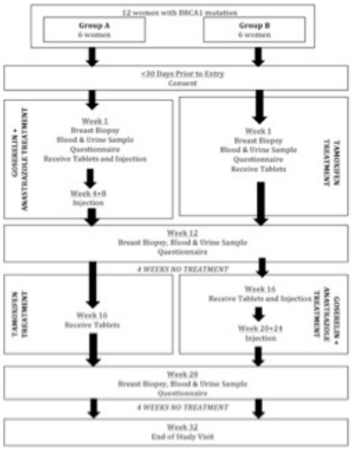

Figure 1: CIBRAC trial schema

338x190mm (54 x 54 DPI)

Reporting checklist for protocol of a clinical trial.

Based on the SPIRIT guidelines.

Instructions to authors

Complete this checklist by entering the page numbers from your manuscript where readers will find each of the items listed below.

Your article may not currently address all the items on the checklist. Please modify your text to include the missing information. If you are certain that an item does not apply, please write "n/a" and provide a short explanation.

Upload your completed checklist as an extra file when you submit to a journal.

In your methods section, say that you used the SPIRIT reporting guidelines, and cite them as:

Chan A-W, Tetzlaff JM, Altman DG, Laupacis A, Gøtzsche PC, Krleža-Jerić K, Hróbjartsson A, Mann H, Dickersin K, Berlin J, Doré C, Parulekar W, Summerskill W, Groves T, Schulz K, Sox H, Rockhold FW, Rennie D, Moher D. SPIRIT 2013 Statement: Defining standard protocol items for clinical trials. Ann Intern Med. 2013;158(3):200-207

		Reporting Item	Page Number
Title	#1	Descriptive title identifying the study design, population, interventions, and, if applicable, trial acronym	1
Trial registration	#2a	Trial identifier and registry name. If not yet registered, name of intended registry	1
Trial registration: data set	#2b	All items from the World Health Organization Trial Registration Data Set	21
Protocol version	#3	Date and version identifier	1
Funding	#4	Sources and types of financial, material, and other support	14
Roles and responsibilities: contributorship	#5a	Names, affiliations, and roles of protocol contributors	1,14
Roles and responsibilities:	#5b	Name and contact information for the trial sponsor	14

For peer review only

sponsor contact information			
Roles and responsibilities: sponsor and funder	#5c	Role of study sponsor and funders, if any, in study design; collection, management, analysis, and interpretation of data; writing of the report; and the decision to submit the report for publication, including whether they will have ultimate authority over any of these activities	14
Roles and responsibilities: committees	#5d	Composition, roles, and responsibilities of the coordinating centre, steering committee, endpoint adjudication committee, data management team, and other individuals or groups overseeing the trial, if applicable (see Item 21a for data monitoring committee)	10
Background and rationale	#6a	Description of research question and justification for undertaking the trial, including summary of relevant studies (published and unpublished) examining benefits and harms for each intervention	4-5
Background and rationale: choice of comparators	#6b	Explanation for choice of comparators	4-5
Objectives	#7	Specific objectives or hypotheses	17
Trial design	#8	Description of trial design including type of trial (eg, parallel group, crossover, factorial, single group), allocation ratio, and framework (eg, superiority, equivalence, non-inferiority, exploratory)	6, and Figure 1.
Study setting	#9	Description of study settings (eg, community clinic, academic hospital) and list of countries where data will be collected. Reference to where list of study sites can be obtained	6
Eligibility criteria	#10	Inclusion and exclusion criteria for participants. If applicable, eligibility criteria for study centres and individuals who will perform the interventions (eg, surgeons, psychotherapists)	17
Interventions: description	#11a	Interventions for each group with sufficient detail to allow replication, including how and when they will be administered	6

For peer review only

Interventions: modifications	#11b	Criteria for discontinuing or modifying allocated interventions for a given trial participant (eg, drug dose change in response to harms, participant request, or improving / worsening disease)	6,7
Interventions: adherance	#11c	Strategies to improve adherence to intervention protocols, and any procedures for monitoring adherence (eg, drug tablet return; laboratory tests)	7
Interventions: concomitant care	#11d	Relevant concomitant care and interventions that are permitted or prohibited during the trial	7
Outcomes	#12	Primary, secondary, and other outcomes, including the specific measurement variable (eg, systolic blood pressure), analysis metric (eg, change from baseline, final value, time to event), method of aggregation (eg, median, proportion), and time point for each outcome. Explanation of the clinical relevance of chosen efficacy and harm outcomes is strongly recommended	17
Participant timeline	#13	Time schedule of enrolment, interventions (including any run-ins and washouts), assessments, and visits for participants. A schematic diagram is highly recommended (see Figure)	6, and Figure 1
Sample size	#14	Estimated number of participants needed to achieve study objectives and how it was determined, including clinical and statistical assumptions supporting any sample size calculations	9
Recruitment	#15	Strategies for achieving adequate participant enrolment to reach target sample size	6
Allocation: sequence generation	#16a	Method of generating the allocation sequence (eg, computer-generated random numbers), and list of any factors for stratification. To reduce predictability of a random sequence, details of any planned restriction (eg, blocking) should be provided in a separate document that is unavailable to those who enrol participants or assign interventions	6
Allocation concealment	#16b	Mechanism of implementing the allocation sequence (eg, central telephone; sequentially numbered, opaque, sealed	See note 1

For peer review only

mechanism		envelopes), describing any steps to conceal the sequence until interventions are assigned	
Allocation: implementation	#16c	Who will generate the allocation sequence, who will enrol participants, and who will assign participants to interventions	6
Blinding (masking)	#17a	Who will be blinded after assignment to interventions (eg, trial participants, care providers, outcome assessors, data analysts), and how	See note 2
Blinding (masking): emergency unblinding	#17b	If blinded, circumstances under which unblinding is permissible, and procedure for revealing a participant's allocated intervention during the trial	N/A - non-blinded study
Data collection plan	#18a	Plans for assessment and collection of outcome, baseline, and other trial data, including any related processes to promote data quality (eg, duplicate measurements, training of assessors) and a description of study instruments (eg, questionnaires, laboratory tests) along with their reliability and validity, if known. Reference to where data collection forms can be found, if not in the protocol	10
Data collection plan: retention	#18b	Plans to promote participant retention and complete follow-up, including list of any outcome data to be collected for participants who discontinue or deviate from intervention protocols	See note 3
Data management	#19	Plans for data entry, coding, security, and storage, including any related processes to promote data quality (eg, double data entry; range checks for data values). Reference to where details of data management procedures can be found, if not in the protocol	See note 4
Statistics: outcomes	#20a	Statistical methods for analysing primary and secondary outcomes. Reference to where other details of the statistical analysis plan can be found, if not in the protocol	9
Statistics: additional analyses	#20b	Methods for any additional analyses (eg, subgroup and adjusted analyses)	9
Statistics: analysis population and missing data	#20c	Definition of analysis population relating to protocol non-adherence (eg, as randomised analysis), and any statistical methods to handle missing data (eg, multiple	9

For peer review only

		imputation)	
Data monitoring: formal committee	#21a	Composition of data monitoring committee (DMC); summary of its role and reporting structure; statement of whether it is independent from the sponsor and competing interests; and reference to where further details about its charter can be found, if not in the protocol. Alternatively, an explanation of why a DMC is not needed	10
Data monitoring: interim analysis	#21b	Description of any interim analyses and stopping guidelines, including who will have access to these interim results and make the final decision to terminate the trial	10
Harms	#22	Plans for collecting, assessing, reporting, and managing solicited and spontaneously reported adverse events and other unintended effects of trial interventions or trial conduct	7
Auditing	#23	Frequency and procedures for auditing trial conduct, if any, and whether the process will be independent from investigators and the sponsor	10
Research ethics approval	#24	Plans for seeking research ethics committee / institutional review board (REC / IRB) approval	11
Protocol amendments	#25	Plans for communicating important protocol modifications (eg, changes to eligibility criteria, outcomes, analyses) to relevant parties (eg, investigators, REC / IRBs, trial participants, trial registries, journals, regulators)	19
Consent or assent	#26a	Who will obtain informed consent or assent from potential trial participants or authorised surrogates, and how (see Item 32)	6/7
Consent or assent: ancillary studies	#26b	Additional consent provisions for collection and use of participant data and biological specimens in ancillary studies, if applicable	N/A
Confidentiality	#27	How personal information about potential and enrolled participants will be collected, shared, and maintained in order to protect confidentiality before, during, and after the trial	See note 5
Declaration of	#28	Financial and other competing interests for principal	14

For peer review only

interests		investigators for the overall trial and each study site	
Data access	#29	Statement of who will have access to the final trial dataset, and disclosure of contractual agreements that limit such access for investigators	See note 6
Ancillary and post trial care	#30	Provisions, if any, for ancillary and post-trial care, and for compensation to those who suffer harm from trial participation	N/A
Dissemination policy: trial results	#31a	Plans for investigators and sponsor to communicate trial results to participants, healthcare professionals, the public, and other relevant groups (eg, via publication, reporting in results databases, or other data sharing arrangements), including any publication restrictions	11
Dissemination policy: authorship	#31b	Authorship eligibility guidelines and any intended use of professional writers	11
Dissemination policy: reproducible research	#31c	Plans, if any, for granting public access to the full protocol, participant-level dataset, and statistical code	11
Informed consent materials	#32	Model consent form and other related documentation given to participants and authorised surrogates	See note 7
Biological specimens	#33	Plans for collection, laboratory evaluation, and storage of biological specimens for genetic or molecular analysis in the current trial and for future use in ancillary studies, if applicable	See note 8

Author notes

1. N/A (non-blinded study)
2. N/A (non-blinded study)
3. Outlined in CIBRAC Data Management Plan
4. Outlined in CIBRAC Data Management Plan
5. Outlined in CIBRAC Data Management Plan
6. Outlined in CIBRAC Data Management Plan
7. N/A - in Trial Master File

For peer review only

8. N/A - in CIBRAC Laboratory Manual

The SPIRIT checklist is distributed under the terms of the Creative Commons Attribution License CC-BY-ND 3.0. This checklist was completed on 20. March 2018 using http://www.goodreports.org/, a tool made by the EQUATOR Network in collaboration with Penelope.ai

For peer review only

BMJ Open

Chemoprevention in BRCA1 mutation carriers (CIBRAC): Protocol for an open allocation crossover feasibility trial assessing mechanisms of chemoprevention with Goserelin and Anastrazole versus Tamoxifen and acceptability of treatment.

Journal:	*BMJ Open*
Manuscript ID	bmjopen-2018-023115.R1
Article Type:	Protocol
Date Submitted by the Author:	24-Sep-2018
Complete List of Authors:	Campbell, Aideen; Queen's University Belfast, Centre for Cancer Research and Cell Biology<br>Morris, Melanie; Belfast Health and Social Care Trust, Northern Ireland Cancer Trials Centre<br>Gallagher, Rebecca; Belfast Health and Social Care Trust, Northern Ireland Cancer Trials Centre<br>Boyd, Ruth; Belfast Health and Social Care Trust, Northern Ireland Cancer Trials Centre<br>Carson, Hazel; BRCALink NI<br>Harkin, D. Paul; Queen's University Belfast, Centre for Cancer Research and Cell Biology<br>Wielogorska, Ewa; Queen's University Belfast, Institute for Global Food Security<br>Elliott, Christopher; Queen's University Belfast, Institute for Global Food Security<br>Savage, Kienan; Queen's University Belfast, Centre for Cancer Research and Cell Biology<br>McIntosh, Stuart; Queen's University Belfast, Centre for Cancer Research and Cell Biology
**Primary Subject Heading**:	Genetics and genomics
Secondary Subject Heading:	Oncology, Surgery
Keywords:	Cancer genetics < GENETICS, PREVENTIVE MEDICINE, Breast surgery < SURGERY

SCHOLARONE™
Manuscripts

BMJ Open Submission

Title Page

Study title:

Chemoprevention in BRCA1 mutation carriers (CIBRAC): Protocol for an open allocation crossover feasibility trial assessing mechanisms of chemoprevention with Goserelin and Anastrazole versus Tamoxifen and acceptability of treatment.

Authors:

A. M. Campbell[1], M. Morris[2], R. Gallagher[2], R. Boyd[2], H. Carson[3], D. P. Harkin[1], E. Wielogorska[4], C. Elliott[5], K. I. Savage[1¶], S. A. McIntosh[1¶*]

Affiliations:

1. Centre for Cancer Research and Cell Biology, Queen's University Belfast, Lisburn Road, Belfast, BT9 7AE.
2. Northern Ireland Cancer Trials Network, East Podium, C Floor, Belfast City Hospital, Lisburn Road, Belfast, BT9 7AB.
3. BRCALink NI, 5 Spa Grange, Ballynahinch, BT24 8PD.
4. School of Pharmacy, Queen's University Belfast, David Keir Building, 18-30 Malone Road, Belfast, BT9 5BN.
5. Institute for Global Food Security, Advanced ASSET Centre, School of Biological Sciences, Queen's University Belfast.

¶ These authors contributed equally

*Corresponding author: S. A. McIntosh (s.mcintosh@qub.ac.uk)

Keywords: BRCA1, oestrogen, chemoprevention, LHRHa, aromatase inhibitor, SERM, anastrazole, goserelin, tamoxifen.

Trial Registration: EudraCT: 2016-001087-11

Protocol version 4.0, 11/09/2017

For peer review only

Abstract

Introduction: BRCA1 mutation carriers have a significant lifetime risk of breast cancer, with their primary risk-reduction option being bilateral mastectomy. Preclinical work from our laboratory demonstrated that in BRCA1-deficient breast cells, oestrogen and its metabolites are capable of driving DNA damage and subsequent genomic instability, which are well-defined early events in BRCA1-related cancers. Based on this, we hypothesise that a chemopreventive approach which reduces circulating oestrogen levels may reduce DNA damage and genomic instability, thereby providing an alternative to risk-reducing surgery.

Methods and Analysis: 12 premenopausal women with pathogenic BRCA1 mutations and no previous risk-reducing surgery will be recruited from Family History Clinics. Participants will be allocated 1:1 to two arms. All will undergo baseline breast biopsies, blood and urine sampling and quality of life (QOL) questionnaires. Group A will receive goserelin 3.6mg/28 days by subcutaneous injection, plus oral anastrazole 1mg/day, for 12 weeks. Group B will receive oral tamoxifen 20mg/day for 12 weeks. Following treatment, both groups will provide repeat biopsies, blood and urine samples, and questionnaires. Following a one-month washout period, the groups will cross over; Group A receiving tamoxifen and Group B goserelin and anastrazole for a further 12 weeks. After treatment, biopsies, blood and urine samples, and questionnaires will be repeated. DNA damage will be assessed in core biopsies, whilst blood and urine samples will be used to measure oestrogen metabolite and DNA-adduct levels.

Ethics and Dissemination: This study has ethical approval from the Office of Research Ethics Committees Northern Ireland (16/NI/0055) and MHRA approval (Reference: 32485/0032/001-0001). The IMPs used in this trial are licensed and in common use, with well-documented safety information. Dissemination of results will be via high-impact journals and relevant national/international conferences. A copy of results will be offered to participants and be made available to patient support groups.

Trial Registration: EudraCT: 2016-001087-11

For peer review only

For peer review only - http://bmjopen.bmj.com/site/about/guidelines.xhtml

Article summary

Strengths and limitations of this study:

- Solid underpinning pre-clinical data generated within our laboratory
- Extensive patient and public involvement in study design and development
- Oestrogen and oestrogen metabolite levels assessed using established, highly sensitive mass spectrometry-based methodology
- Small sample size, which means that translational endpoints are exploratory
- Lack of qualitative intervention to support recruitment and investigate reasons for declining study participation
- DNA damage is not currently a validated biomarker for cancer risk

For peer review only

For peer review only - http://bmjopen.bmj.com/site/about/guidelines.xhtml

Introduction

Women with a germline BRCA1 mutation have up to an 85% lifetime risk of developing breast cancer by age 70, with the majority of these women developing triple negative disease [1, 2]. Intriguingly, a number of retrospective studies, including a large meta-analysis, have demonstrated that risk-reducing oophorectomy significantly reduces the risk of developing breast cancer in this population by up to 50% [3, 4]. In contrast, more recent studies, including a prospective study, contradict this, suggesting some protective effect in BRCA2 but not BRCA1 mutation carriers[5, 6]. However, these studies have limited follow-up (mean follow-up period 5.6 and 3.2 years respectively), and it is clear from chemoprevention studies carried out in large populations of women at increased risk that a limited chemopreventive effect is seen before 5 years, with significantly greater protective effects seen beyond 10 years[7]. Furthermore, oophorectomy in a mammary-specific BRCA1 knockout mouse model reduces mammary tumour formation as compared with non-oophorectomised mice[8]. Taking this together, the evidence suggests that oophorectomy may confer a protective effect in BRCA1 mutation carriers, however, long-term prospective data is required to support this. Currently, risk-reducing mastectomy is the mainstay of preventative treatment offered to BRCA1 mutation carriers, which is effective but carries significant associated costs in terms of physical and psychological morbidity and healthcare spending [9-11]. Aside from risk-reducing mastectomy, the only other risk-reduction strategy available to these women is chemoprevention.

In the United Kingdom, the National Institute for Health and Care Excellence (NICE, all abbreviations defined in List of Abbreviations, Supplementary File 1) recommends the selective oestrogen receptor modulators (SERMs) tamoxifen and raloxifene for use in women at high-risk of developing breast cancer, on the basis that they have been demonstrated to reduce the incidence of oestrogen-receptor positive tumours [12]. However, evidence for the benefit of tamoxifen as a chemopreventive agent in BRCA1 mutation carriers is conflicting. One small study showed no benefit from tamoxifen in reducing breast cancer risk in BRCA1 mutation carriers, although it has been suggested that tamoxifen use in BRCA1 mutation carriers with breast cancer may reduce the incidence of contralateral tumours [13, 14]. In one case-control study, short term (2 year) tamoxifen use was as protective for contralateral breast cancer as a longer (5 year) course in BRCA1 and BRCA2 mutation carriers, suggesting some potential chemopreventive efficacy [15].

However, given that the majority of tumours that develop in BRCA1 mutation carriers are oestrogen receptor (ER) negative, it seems counterintuitive that SERMs will reduce breast cancer risk, given that no benefit in ER negative tumours has been shown in the chemoprevention setting with tamoxifen [12]. Clinical trial data from the IBIS-II study suggests a reduction in breast cancer risk using aromatase inhibitors in women with increased risk [16]. However, there is no direct evidence to support a benefit for this approach in BRCA1 mutation carriers.

Clearly oestrogen plays a key role in tumourigenesis in these patients, as tumours develop preferentially in the oestrogen-rich tissues of the breast and ovary.

Preclinical data from our laboratory demonstrates that oestrogen and oestrogen metabolites cause DNA double strand breaks (DSBs) in ER negative breast cells (normal and cancer cell lines as well as primary breast progenitor cells isolated from patients), and that BRCA1 is necessary for repression and repair of these DSBs [17]. The implication of these findings is that in BRCA1-deficient breast cells, exposure to oestrogen and its metabolites is capable of driving genomic instability, a well-defined early event in BRCA1-related cancer development [18, 19].

Taken with evidence from clinical trials, which demonstrate no benefit from SERMs in reducing the incidence of ER negative tumours, this suggests that SERMs are unlikely to be effective chemopreventive agents in BRCA1 mutation carriers (who are more likely to develop ER negative tumours). Our data suggests that a chemopreventive approach which lowers circulating oestrogen levels and reduces exposure to oestrogen metabolites within breast tissue may be more successful by removing this driver of genomic instability. Whilst DNA damage is not at present a validated biomarker

For peer review only - http://bmjopen.bmj.com/site/about/guidelines.xhtml

of cancer risk in mutation carriers, as described earlier, genomic instability is a well-characterised early hallmark of BRCA1-associated breast tumours. Consistent with this, mice harbouring specific point mutations within BRCA1 affecting its DNA repair function whilst conserving other protein functions (E3 ligase, transcriptional activity, etc.) develop tumours [20]. This supports the link between DNA damage, genomic instability and tumourigenesis in BRCA1-deficient breast cells.

In post-menopausal women (or pre-menopausal women who have undergone oophorectomy), the use of an aromatase inhibitor may be considered. In premenopausal women with intact ovaries, the induction of reversible ovarian suppression using a luteinising hormone releasing hormone agonist (LHRHa) in combination with an aromatase inhibitor (AI), to suppress subsequent aromatase-mediated oestrogen production, may be effective.

Progesterone signaling has also recently been implicated in BRCA1-related tumourigenesis, with the TNF-superfamily member RANK and its corresponding ligand RANKL functioning as key factors in this pathway and potential targets for chemoprevention [21]. Importantly, LHRHa suppression of the hypothalamic-pituitary axis will also result in decreased progesterone levels, which will consequently reduce progesterone-dependent RANK/RANKL signaling. Additionally, expression of the progesterone receptor (PR), a critical mediator of the progesterone/RANK/RANKL pathway, is itself driven by oestrogen signaling through the ER. Taken together, the suppression of oestrogen is likely to provide chemopreventive potential, similar to or surpassing that demonstrated through RANK/RANKL suppression.

Although LHRHa, AIs and SERMs are used in breast cancer and fertility treatments, there is no published data in the context of BRCA1-specific chemoprevention where the intention is to prevent ER negative breast cancer.

It is known that compliance and acceptability of these agents in the adjuvant setting of breast cancer treatment is good, albeit with a high frequency of side effects [22].

The compliance of women taking SERMs for chemoprevention is sub-optimal, with approximately 40% of women unable to complete 5 years of therapy because of side effects [23]. Therefore, compliance with LHRHa in combination with an aromatase inhibitor in the chemopreventive setting must be assessed and compared with SERM data as an important consideration in the feasibility of use in this context. In line with this, this study aims to inform future tolerability and compliance evaluation in a cohort who may require long durations of treatment and have a long life expectancy.

If the results from this study demonstrate that these treatments are a feasible and tolerable strategy, and that DNA damage is reduced through reduced levels of circulating oestrogen, this would provide data to support the proposed mechanism of oestrogen-driven tumourigenesis in BRCA1 mutation carriers. Furthermore, this may provide evidence to support further studies evaluating the use of this chemopreventive strategy in BRCA1 mutation carriers. Nevertheless, this would require a multicentre international collaborative study, which presents significant challenges. Undoubtedly the treatment regimen proposed within this study has a clear side-effect profile, the tolerability and acceptability of which will be addressed in this study. Currently, a number of groups are investigating inhibition of progesterone and/or RANK/RANKL signaling as an alternative chemopreventive strategy in BRCA1 carriers, which may present a more tolerable approach[24]. However, a recent study using a well-characterised mouse model of BRCA1-associated breast cancer suggests that oestrogen is the predominant driver of tumourigenesis rather than progesterone[25]. Given that RANK/RANKL signaling is progesterone-dependent, the efficacy of these progesterone and RANK/RANKL targeted chemopreventive approaches remains unproven. In light of this, continued exploration of oestrogen suppression in this cohort is justified in order to maximise the repertoire of potential chemopreventive options in this area of unmet clinical need.

For peer review only - http://bmjopen.bmj.com/site/about/guidelines.xhtml

Methods/Analysis

The aims, design and setting of the study

The principal aim of this research is to establish if these treatments are tolerable, and to evaluate patient compliance. Secondary aims include tolerability of treatments and quality of life.
As exploratory objectives, this research seeks to not only confirm the link between oestrogen exposure and DSBs and generate further insight into how oestrogen and its metabolites affect tumourigenic transformation, but also to generate the preliminary data necessary to support development of larger clinical study to evaluate chemoprevention in BRCA1 mutation carriers.

Study aims and objectives are summarised in Table 1.

Design

A schematic overview of the trial is depicted in Figure 1.
The crossover design allows comparison of outcomes from both treatments to the participant baseline, thus each participant acts as their own control for both treatment arms.
Blinding or masking is not achievable given the nature of administration of goserelin (subcutaneous implant).
Given that each participant will receive both treatments without blinding, randomization was felt unnecessary and alternate allocation of treatment groups in order of recruitment is adequate.
The setting of the trial in tertiary care (Family History Breast Clinics) reflects the main location for the ongoing care and management of this cohort.

Selection of participants

Participants will be recruited from Family History Breast Clinics within Northern Ireland as identified by clinicians and by posters and publicity regarding the trial presented and distributed at relevant public engagement events.
The participant inclusion and exclusion criteria are outlined in Table 2. The Chief Investigator (or appropriately trained delegate) will confirm eligibility against the inclusion/exclusion criteria.

Description of Interventions

Goserelin and Anastrazole Treatment
Participants will receive goserelin 3.6mg by subcutaneous injection every 28 days for 12 weeks, simultaneously receiving oral anastrazole 1mg per day for 12 weeks.

Tamoxifen Treatment
Participants will receive oral tamoxifen 20mg per day for 12 weeks.

Washout Period
Upon completion of the first treatment arm, participants will have a 4-week period of no treatment to allow washout of active substances, before commencing second treatment arm.

Description of Processes

Procedure for obtaining informed consent and trial arm allocation
Participants will receive an adequate explanation of the objectives, methods, potential risks and anticipated benefits of the study. They will be given a current, ethically approved participant information sheet (PIS) for their consideration. Following this, informed consent will be obtained once women have had a minimum of 24 hours to consider trial entry and the opportunity to ask further questions. Following consent, participants will be provided with a copy of the signed consent form,

For peer review only - http://bmjopen.bmj.com/site/about/guidelines.xhtml

with a copy filed in the patient notes, and the original retained in the site file, available for inspection if required.
No protocol required assessments will be conducted until the consent form has been signed and dated by both participant and investigator.
Participants will be provided the option to allow the use of blood samples, other body fluids, and tissues obtained during testing, operative procedures, or other standard medical practices for further research purposes.
Participants will be alternately allocated to treatment arms.

Replacement of participants
In the case of a participant request for withdrawal or failure to collect biological samples, that participant will be withdrawn from the study and a new participant enrolled into the treatment arm the original participant has been retracted from.

Percentage Consenting to Trial Entry
Records will be kept of all the Patient Information Sheets distributed to allow calculation of percentage of patients consenting to trial entry.

Compliance Measurement
Participants will receive a medication diary card for each oral treatment at the same time the tablets (Anastrazole for Arm A and Tamoxifen for Arm B) are issued to track compliance.

Concomitant medication:
Participants must be instructed not to take any medications (including herbal and over-the-counter remedies) during the study without prior consultation with the investigator.
Any hormonal treatments (including all forms of hormonal contraception) are prohibited.
Concomitant medications must be recorded in the patients' notes at the time of initial consultation and any amendments documented at subsequent visits.

Quality of Life Assessment
Questionnaires will be completed on Day 1 (baseline), Day 84 (+/-3days, following the end of first arm) and Day 196 (+/-3days following the end of second arm).
Two questionnaires will be used to assess health-related Quality of Life (QOL):

1. EORTC QLQ-C30 questionnaire assessing quality of life
2. EORTC QLQ-BR23 questionnaire assessing treatment side effects and sexual dysfunction

Recording of Adverse Events
An Adverse Event (AE) is any untoward medical occurrence (including deterioration of a pre-existing medical condition) in a subject administered an Investigational Medicinal Product (IMP). The event does not necessarily have a causal relationship with treatment or usage.
An Adverse Reaction (AR) is an AE that is suspected as having a causal relationship to the IMP.
Each AE and its causality, severity, and expectedness are assessed by the chief investigator (or nominated representative) and recorded in the patient notes.
All IMPs in this study are licensed with well-defined safety profiles. As such, we will only record data relating to ARs in the case report form (CRF).
If the event is classified as serious (SAE) the reporting procedures as per Belfast Health and Social Care Trust will also be followed.
Follow-up will continue until all the necessary safety data for the event has been gathered and until the AR or SAE has resolved, returned to baseline or stabilised.

For peer review only - http://bmjopen.bmj.com/site/about/guidelines.xhtml

For peer review only

Pregnancy Testing

Participants will be asked to provide a non-sterile urine sample (the initial stream) in a clean, non-sterile receiver. The sample will be used to perform a urinary HCG test in the clinic following manufacturer's SOP.

Pregnancy testing will be performed twice:

1. At the time of assessment of eligibility for the trial following consent, prior to the first arm treatment commencement
2. On Day 112 +/-3 (week 16), prior to starting the second treatment arm

Core Biopsies

Biopsy samples will be collected as standard using a 14G core biopsy needle under ultrasound guidance in the Department of Radiology at Belfast City Hospital. Two core biopsies will be sharply dissected into at least 4 smaller samples (approx. 5mm^3 each) and placed in individual Eppendorf tubes, then snap frozen with liquid nitrogen and stored for later use. Two core biopsies will be placed in individual formalin-containing sample pots, after which they will be Formalin Fixed Paraffin Embedded (FFPE) as per standard operating procedures within the Belfast Trust.

Core biopsies will be taken on Day 1 (baseline), Day 84 (+/-3days following end of first arm) and Day 196 (+/-3days following end of second arm).

Histopathological Analysis

One FFPE sample will be assessed by a histopathologist to ensure adequate sampling of glandular tissue.

Comet Assays

Two frozen biopsy specimens will be used to perform tissue-based comet assays to assess DNA damage.

Immunohistochemistry

One FFPE core biopsy sample will be sectioned and stained with antibodies against 53BP1 and γH2AX using conventional and fluorescent immunohistochemistry to assess DNA DSBs in these samples.

Additional sections will also be stained for RANK and RANKL as well as Ki67 using immunohistochemistry.

Mass Spectrometry

Two frozen biopsy specimens will be used to examine concentrations of oestrogen metabolites 2-hydroxyestradiol (2-OHE$_2$) and 4-hydroxyestradiol (4-OHE$_2$), using UPLC-MS/MS.

Serum Samples

Serum sampling will be performed on Day 1 (baseline), Day 84 (+/-3 days following end of first arm) and Day 196 (+/-3 days following, end of second arm). Participants will undergo venepuncture with blood samples collected in two silica (Clot Activator) coated 10ml blood sample tubes. After the minimum recommended clotting time (60 minutes), blood samples will be centrifuged to allow separation of serum from blood cells, with the serum stored at -80°C in 1ml aliquots in RNAse free tubes. Following sample accrual, samples will be thawed and serum 2-OHE$_2$ and 4-OHE$_2$ levels and levels of depurinating DNA adducts (common types of DNA damage caused by oestrogen metabolites) will be measured using UPLC-MS/MS.

Urine Samples

Urine sampling will be performed on Day 1 (baseline), Day 84 (+/-3 days following, end of first arm) and Day 196 (+/-3 days following, end of second arm). Mid-stream urine samples will be collected in sterile containers. Within 2 hours of collection samples will be centrifuged to separate debris from the

For peer review only - http://bmjopen.bmj.com/site/about/guidelines.xhtml

urine and then stored at -80°C in 500μl aliquots in RNAse free tubes. Following sample accrual. Samples will be thawed and urinary 2-OHE$_2$ and 4-OHE$_2$ levels and levels of depurinating DNA adducts will be measured using UPLC-MS/MS.

Whole Blood Sampling

Each participant will provide one whole blood sample at one time point in the study. This will be performed at baseline (Day 1), unless the patient is already enrolled and receiving treatment, in which case the whole blood sampling will be performed at the end of the treatment arm that they are currently receiving (Day 84 +/-3d or Day 196 +/-3d).

The whole blood samples will be collected at the same venepuncture session as serum sampling. Blood will be collected into one EDTA coated tube and frozen/stored at -80°C in 1ml aliquots. Samples will be retained for future use including DNA extraction.

Timeframe for interventions and assessments is summarized in Table 3.

Comparisons

Each participant acts as her own control. Results from each individual's set of samples (core biopsies, serum and urine) and questionnaires, taken at baseline end of first arm and end of second arm will be compared. Results will not be compared between patients.

Statistical Analysis

This study is primarily aimed to determine acceptability and tolerability of the proposed chemopreventive treatments. Therefore, no formal sample size calculation has been carried out, as the laboratory end-points are all exploratory.

For these reasons, no formal statistical analysis is planned. Nonetheless, descriptive statistics will be used to summarise the principal and experimental findings of the study.

Laboratory analysis:

The proposed translational studies include performing comet assays on fresh frozen breast core biopsies at baseline and after each treatment arm. The comet assay protocol has been optimized on breast cell cultures as well as fresh frozen breast biopsy samples using neutral lysis techniques with automated scoring performed on a high content screening system. FFPE breast core biopsy blocks are created at the same time-points as fresh-frozen samples. Formation of γ-H2AX in response to DNA double stranded breaks (DSBs) provides the basis for a sensitive immunohistochemical assay of DNA damage in human biopsies and has been optimised in FFPE breast samples with simultaneous staining for 53BP1, which localises to DNA strand breaks, with foci numbers proportional to DNA damage levels. Based on our hypothesis, the anticipated decrease in measurable DNA damage in response to oestrogen suppression would support the use of this strategy as a chemoprevention option in BRCA1 mutation carriers.

Further FFPE sections will be analysed for Ki67, RANK and RANKL expression to assess the effect of treatment on these markers of proliferation (Ki67) and progesterone signalling (RANK/RANKL). As the LHRHa used to suppress oestrogen will also result in progesterone suppression, it is expected that RANK and RANKL expression will also decrease. Given that inhibition of RANK has been shown to reduce breast cell proliferation in BRCA1 mutation carriers, it is possible that LHRHa treatment might be an alternative way of down regulating this pathway and potentially decreasing the development of highly proliferative cells with grossly aberrant DNA repair observed in BRCA1 precancerous tissues with high levels of RANK/RANKL expression [15].

Oestrogen metabolite levels in serum and urine will be measured using ultra performance liquid chromatography tandem mass spectrometry (UPLC-MS/MS) and have been optimised on healthy volunteer samples with permission from Queen's University Ethics Committee.

For peer review only - http://bmjopen.bmj.com/site/about/guidelines.xhtml

Patient and public involvement:

BRCA1 and BRCA2 mutation carriers were involved throughout the design process via the Northern Ireland BRCA mutation carrier support group, BRCALink NI. The study design was finalized with input from a BRCA patient advocate focus group, where the consensus was that inclusion of an active control treatment was more acceptable than placebo. Women within the group had experienced tamoxifen therapy as part of cancer treatment and deemed potential side effects acceptable in the context of risk reduction. The BRCA mutation carrier cohort in Northern Ireland is overwhelmingly enthusiastic about chemoprevention clinical trials, resulting in a highly motivated patient group keen to contribute to future solutions to BRCA-related breast cancer risk. In keeping with this, the trial management group (TMG) for this study includes a BRCA1 mutation carrier.

Data Collection and Management:

The Investigator and study site staff will ensure that data collected on each subject is recorded on the Case Report Form (CRF) as accurately and completely as possible. Data management processes will be fully defined within the Data Management Plan for the study, held within the trial master file (TMF).

Trial Management

Trial Management Group (TMG)
The TMG will consist of an independent chair, the Chief Investigator, Co-investigators and clinical research nurse as well as other relevant committee members as defined within the TMG charter. The TMG will provide overall supervision of the trial on behalf of the trial funder and sponsors, and ensure that the trial is conducted according to Good Clinical Practice guidelines. The TMG will focus on progress of the trial, adherence to trial protocol, and patient safety. The TMG will report to the BHSCT CTIMP Oversight Committee.
Meetings frequency will be defined within the TMG Charter but as a minimum will meet at least quarterly.

Trial Steering Committee (TSC)
The Belfast Health and Social Care Trust CTIMP Oversight Committee, which meets quarterly, acts as the TSC for CIBRAC.

Independent Data Monitoring Committee (IDMC)
After review by the Sponsor, it was not considered that an IDMC was required for this trial due to its low risk nature, in that the IMPs are not novel agents, and are licensed agents with a well-defined safety profile.

Monitoring
The Sponsor has appointed the Clinical Research Monitoring Service within the Northern Ireland Clinical Trials Unit to undertake on site monitoring. An initial monitoring visit was carried out prior to the release of the final permissions letter by the Sponsor. The first interim monitoring visit will take place within 6 months of the first patient being recruited. Further monitoring visits will take place approximately every 6 months from the date of the first visit. A close-out visit will occur once the last patient visit has been completed. A trial monitoring plan is available within the TMF.

The SPIRIT checklist has been used in writing this report[26].

For peer review only - http://bmjopen.bmj.com/site/about/guidelines.xhtml

Ethics and dissemination

Ethics: The Health and Social Care Research Ethics Committee B (HSC REC B), as part of the Office for Research Ethics Committees Northern Ireland (ORECNI), awarded ethical approval for this study (REC Reference:16/NI/0055, 21/4/16). A summary of amendments is given in Table 4.

The Investigational Medical Products used in this study are all licensed agents, which are in common use in the treatment of breast cancer. They therefore have well-defined safety profiles available.
The women offered the opportunity to participate in this study are not cancer patients, but rather are healthy pre-menopausal women who carry a pathogenic mutation in the BRCA1 gene. Although this is effectively a healthy volunteer study, it was felt it would not be appropriate to offer incentives for participation, although travelling expenses are covered for trial hospital visits.
There was extensive patient and public involvement in the study design and development, to ensure that the proposed treatments and study procedures would be acceptable to patients.

Trial registration: CIBRAC is registered as a clinical trial of an interventional medical product on the European Clinical Trials Database, as EudraCT: 2016-001087-11. Trial registration data are provided in Table 5.

Dissemination: Academic dissemination of the study will be via publication in peer-reviewed journals in the relevant field (the ICMJE criteria for authorship will be followed), and by presentation at appropriate national and international meetings. These may include meetings such as the biannual Symposium on Hereditary Breast and Ovarian Cancer in Montreal, Canada. A lay summary of the study results will be available, and all participants will be offered access to a copy of this. Furthermore, this will be shared with our partner PPI organization BRCALink NI and disseminated through their website and social media channels. All mass spectrometry and sequencing data generated from this study and/or the future use of study samples will be deposited in appropriate publically-accessible repositories, with the associated anonymised meta-data.

Word count: 3536 words

For peer review only

For peer review only - http://bmjopen.bmj.com/site/about/guidelines.xhtml

List of abbreviations

See supplementary file.

Declarations

Author's contributions

SMcI and KS generated the study idea and hypothesis. AC was responsible for writing the protocol, supervised by SMcI and KS.
MM, RG, RB and HC contributed to protocol development, writing and revision.
EW, CE and DPH contributed to experimental design and validation. DPH also contributed to protocol development.
SMcI is the Chief Investigator of the study and has overall responsibility for its conduct.
All authors have seen and approved the final manuscript.

Funding statement

This work is supported by Cancer Research UK as part of a Clinical Fellowship award. The funding body have no input into the design of the study, the collection, analysis or interpretation of data, nor into the writing of the manuscript.

Competing interests

The authors declare they have no competing interests.

Acknowledgements

We are grateful for the contribution of our patient advocates in developing the study, particularly Hazel Carson of BRCALink NI.

Trial Sponsor

Belfast Health and Social Care Trust (BHSCT)
Sponsorship Contact Details:
Alison Murphy,
Belfast Health and Social Care Trust
Research & Development Office, Room 2010, 2nd Floor,
King Edward Building,
Royal Hospitals Site,
Grosvenor Road,
Belfast,
BT12 6BA.

Role of sponsor: The study sponsor undertook peer review of the protocol. However, the sponsor had no role in study design, nor will they be involved in data collection, analysis, interpretation of data, report writing or decisions in respect of publication.

For peer review only

References

1. Antoniou, A., et al., *Average risks of breast and ovarian cancer associated with BRCA1 or BRCA2 mutations detected in case Series unselected for family history: a combined analysis of 22 studies.* Am J Hum Genet, 2003. **72**(5): p. 1117-30.
2. Lakhani, S.R., et al., *Prediction of BRCA1 status in patients with breast cancer using estrogen receptor and basal phenotype.* Clin Cancer Res, 2005. **11**(14): p. 5175-80.
3. Eisen, A., et al., *Breast cancer risk following bilateral oophorectomy in BRCA1 and BRCA2 mutation carriers: an international case-control study.* J Clin Oncol, 2005. **23**(30): p. 7491-6.
4. Rebbeck, T.R., N.D. Kauff, and S.M. Domchek, *Meta-analysis of risk reduction estimates associated with risk-reducing salpingo-oophorectomy in BRCA1 or BRCA2 mutation carriers.* J Natl Cancer Inst, 2009. **101**(2): p. 80-7.
5. Kotsopoulos, J., et al., *Bilateral Oophorectomy and Breast Cancer Risk in BRCA1 and BRCA2 Mutation Carriers.* J Natl Cancer Inst, 2017. **109**(1).
6. Heemskerk-Gerritsen, B.A., et al., *Breast Cancer Risk After Salpingo-Oophorectomy in Healthy BRCA1/2 Mutation Carriers: Revisiting the Evidence for Risk Reduction.* J Natl Cancer Inst, 2015. **107**(5).
7. Cuzick, J., et al., *Tamoxifen for prevention of breast cancer: extended long-term follow-up of the IBIS-I breast cancer prevention trial.* Lancet Oncol, 2015. **16**(1): p. 67-75.
8. Bachelier, R., et al., *Effect of bilateral oophorectomy on mammary tumor formation in BRCA1 mutant mice.* Oncol Rep, 2005. **14**(5): p. 1117-20.
9. Ludwig, K.K., et al., *Risk reduction and survival benefit of prophylactic surgery in BRCA mutation carriers, a systematic review.* Am J Surg, 2016. **212**(4): p. 660-669.
10. Hallowell, N., et al., *Looking different, feeling different: women's reactions to risk-reducing breast and ovarian surgery.* Familial Cancer, 2012. **11**(2): p. 215-224.
11. Robertson, S.A., et al., *Resource implications of risk-reducing mastectomy and reconstruction.* (1532-2157 (Electronic)).
12. Cuzick, J., et al., *Selective oestrogen receptor modulators in prevention of breast cancer: an updated meta-analysis of individual participant data.* The Lancet, 2013. **381**(9880): p. 1827-1834.
13. King, M.C., et al., *Tamoxifen and breast cancer incidence among women with inherited mutations in BRCA1 and BRCA2: National Surgical Adjuvant Breast and Bowel Project (NSABP-P1) Breast Cancer Prevention Trial.* Jama, 2001. **286**(18): p. 2251-6.
14. Gronwald, J., et al., *Tamoxifen and contralateral breast cancer in BRCA1 and BRCA2 carriers: an update.* Int J Cancer, 2006. **118**(9): p. 2281-4.
15. Gronwald, J., et al., *Duration of tamoxifen use and the risk of contralateral breast cancer in BRCA1 and BRCA2 mutation carriers.* Breast Cancer Res Treat, 2014. **146**(2): p. 421-7.
16. Cuzick, J., et al., *Anastrozole for prevention of breast cancer in high-risk postmenopausal women (IBIS-II): an international, double-blind, randomised placebo-controlled trial.* Lancet, 2014. **383**(9922): p. 1041-8.
17. Savage, K.I., et al., *BRCA1 deficiency exacerbates estrogen-induced DNA damage and genomic instability.* Cancer Res, 2014. **74**(10): p. 2773-84.
18. Deng, C.X., *Tumorigenesis as a consequence of genetic instability in Brca1 mutant mice.* Mutat Res, 2001. **477**(1-2): p. 183-9.
19. Nik-Zainal, S., et al., *The life history of 21 breast cancers.* Cell, 2012. **149**(5): p. 994-1007.
20. Shakya, R., et al., *BRCA1 tumor suppression depends on BRCT phosphoprotein binding, but not its E3 ligase activity.* Science, 2011. **334**(6055): p. 525-8.
21. Nolan, E., et al., *RANK ligand as a potential target for breast cancer prevention in BRCA1-mutation carriers.* Nat Med, 2016. **22**(8): p. 933-9.

For peer review only - http://bmjopen.bmj.com/site/about/guidelines.xhtml

22. Bernhard, J., et al., *Patient-reported outcomes with adjuvant exemestane versus tamoxifen in premenopausal women with early breast cancer undergoing ovarian suppression (TEXT and SOFT): a combined analysis of two phase 3 randomised trials.* The Lancet Oncology, 2015. **16**(7): p. 848-858.
23. Roetzheim, R.G., et al., *Acceptance and adherence to chemoprevention among women at increased risk of breast cancer.* Breast, 2015. **24**(1): p. 51-6.
24. Kotsopoulos, J., C. Singer, and S.A. Narod, *Can we prevent BRCA1-associated breast cancer by RANKL inhibition?* Breast Cancer Res Treat, 2017. **161**(1): p. 11-16.
25. van de Ven, M., et al., *BRCA1-associated mammary tumourigenesis is dependent on oestrogen rather than progesterone signalling.* J Pathol, 2018.
26. Chan, A.W., et al., *SPIRIT 2013 statement: defining standard protocol items for clinical trials.* Ann Intern Med, 2013. **158**(3): p. 200-7.

For peer review only

Figure 1: CIBRAC trial schema

For peer review only - http://bmjopen.bmj.com/site/about/guidelines.xhtml

Table 1: Trial objectives and endpoints

**Primary Objective**	**Primary Endpoint**
To establish the acceptability of trial treatments as a chemopreventive strategy in BRCA1 mutation carriers and compliance.	Percentage of patients given Patient Information Sheet (PIS) who consent to trial entry.<br>Measurement of compliance with treatment using patient medication cards.
**Secondary Objective**	**Secondary Endpoint**
To establish tolerability of trial treatments and procedures	Measurement of patient Quality of Life using questionnaires at baseline and after completion of each treatment arm.<br>Recording of Adverse Events (AEs) that occur during treatment
**Exploratory Objectives**	**Exploratory Endpoints**
To establish the potential of chemopreventive agents to reduce oestrogen mediated DNA damage in breast tissue in BRCA1 mutation carriers	Measurement of DNA damage in breast tissue using comet assays and immunohistochemical assays at baseline and after completion of each treatment arm.
To establish effect of treatment on oestrogen metabolite levels in breast tissue, urine and serum.	Measurement of breast, blood and urinary oestrogen metabolite levels using UPLC-MS/MS at baseline and after completion of each treatment arm.
To explore the mechanism of oestrogen mediated DNA damage in breast tissue in BRCA1 mutation carriers.	Analysis of breast tissue samples using alternative methods of DNA damage assessment.
To explore the relationship between oestrogen metabolite concentration in serum and urine and DNA damage in breast tissue in BRCA1 mutation carriers.	Analysis of breast tissue, urine and blood samples for proxies of oestrogen levels and DNA damage.
To explore potential biomarkers of chemoprevention efficacy.	Analysis of breast tissue, urine and blood samples for potential biomarkers of chemopreventive efficacy.

For peer review only

For peer review only - http://bmjopen.bmj.com/site/about/guidelines.xhtml

Table 2: Inclusion and exclusion criteria

Inclusion Criteria	Exclusion Criteria
Age >18 years	BRCA1 mutation of uncertain significance
Premenopausal	Personal history of breast or ovarian carcinoma
Known pathogenic BRCA1 mutation	Previous risk-reducing breast or ovarian surgery
Intact ovaries	Post-menopausal status
No previous breast/ovarian carcinoma	Concomitant use of alternative chemoprevention regimens
No other previous malignancy	Concomitant use of other hormonal agents less than one month prior to enrolment
No previous use of chemoprevention	Contraindications to study drug therapies
Willingness to use non-hormonal contraception	Contraindications to breast core biopsies
	Pregnancy or breastfeeding
	Inability to give informed consent
	Having made a decision to proceed with risk reducing surgery

For peer review only

For peer review only - http://bmjopen.bmj.com/site/about/guidelines.xhtml

Table 3: Summary of assessments and interventions

Time Point	<30 days prior to entry	Week 1	Week 4	Week 8	Week 12	Week 16	Week 20	Week 24	Week 28	Week 32 (EOT)
**Enrolment**										
Informed Consent	A+B									
Baseline History	A+B									
Eligibility Screen & Pregnancy Test		A+B				A+B				
Treatment Allocation		A+B								
Conmeds	A+B	A+B	A	A	A+B	A+B	B	B	A+B	A+B
**Interventions**										
Start Tamoxifen (12 weeks)		B				A				
Start Anastrazole (12 weeks)		A				B				
Goserelin Implant Injection		A	A	A		B	B	B		
**Assessments**										
QOL Assessment		A+B			A+B				A+B	
Core Breast Biopsies x3		A+B			A+B				A+B	
Blood samples for oestrogen & metabolite levels		A+B			A+B				A+B	
Blood sample for whole blood		A+B			A+B				A+B	
Urine samples for oestrogen & metabolite levels		A+B			A+B				A+B	
AE assessment	A+B	A+B			A+B	A+B			A+B	A+B

For peer review only

For peer review only - http://bmjopen.bmj.com/site/about/guidelines.xhtml

Table 4: Amendment chronology

Amendment Number	Summary of Amendment	Documents Impacted (and version)	Date sent for classification	Date Classified	Date sent to MHRA	Date of MHRA approval	Date sent to Ethics	Date of Ethics Approval	Date sent to R and D	Date of R and D approval	Effective Date
**1**	contraceptive advice amended	Protocol 1.2 dated 24-May-16	04-Sep-16	Minor 16-Sept-16	N/A	N/A	21-Jun-16	21-Jun-16	04-Sep-16	N/A	N/A
**2**	changes in the priority of existing objectives & endpoints	Protocol2.0 dated 30-Aug-16 PIS ICF 1.1 dated 19-Apr-16	04-Sep-16	Substantial 16-Sept-16	N/A	N/A	23-Sep-16	27-Sep-16	11-Oct-16	19-Jan-17	N/A
**3**	type of blood amended and volume	Protocol 2.1 dated 09-Jan-17	09-Jan-17	Substantial 30-Jan-2017	N/A	N/A	07-Feb-17	01-Mar-17	01-Mar-17	14-Mar-17	14-Mar-17
**Minor 20-Jan-17**	addition of Keith Lowry and Lesley McFaul	Not applicable	N/A	N/A	N/A	N/A	N/A	N/A	20-Jan-17	13-Feb-17	13-Feb-17
**4**	Sample Collection change	Protocol 3.0	04-Apr-17	Substantial 08/05/2017	N/A	N/A	08-May-17	02-Jun-17	10-May-17	14-Jun-17	15-Jun-17
**5**	Letter to potential participants, modify exclusion criteria	Protocol 4.0 dated 11/9/17									
**Letter 1.0 dated 11/09/17**	11-Sept-17	Substantial 21/09/17	N/A	N/A	29/09/17	10-Oct-2017	10-Oct-2017	09-Nov-2017	16-Nov-2017		

For peer review only - http://bmjopen.bmj.com/site/about/guidelines.xhtml

Table 5: Trial registration data

Data category	Information
Primary registry and trial identification number	EudraCT: 2016-001087-11
Date of registration in primary registry	9th March 2016
Sources of monetary or material support	Supported by a Cancer Research UK Clinical Research Fellowship
Sponsor	Belfast Health & Social Care Trust
Contact for public queries	AMC (a.campbell@qub.ac.uk)
Contact for scientific queries	SMcI, KS (s.mcintosh@qub.ac.uk, k.savage@qub.ac.uk)
Public title	CIBRAC: Chemoprevention in BRCA1 mutation carriers
Scientific title	Chemoprevention in BRCA1 mutation carriers (CIBRAC): An open allocation crossover trial assessing mechanisms of chemoprevention of Goserelin and Anastrazole versus Tamoxifen
Countries of recruitment	United Kingdom (Northern Ireland)
Interventions	Tamoxifen 20mg daily (oral)<br>Goserelin 3.6mg every 28 days (sc) plus anastrazole 1mg daily (oral)
Key inclusion criteria	Ages eligible for study ≥18 years<br>Sexes eligible for study: female<br>Known pathogenic germline BRCA1 mutation<br>Pre-menopausal<br>No previous breast or ovarian risk-reducing surgery
Key exclusion criteria	Personal history of breast or ovarian carcinoma<br>Previous risk-reducing breast or ovarian surgery<br>Post-menopausal status
Study type	Interventional<br>Allocation: alternate treatment allocation, non-randomised, non-blinded<br>Primary purpose: prevention<br>Phase II
Date of first enrolment	May 2017
Recruitment status	Recruiting
Primary outcome	Establish the acceptability of trial treatments as a chemopreventive strategy in BRCA1 mutation carriers, as measured by number of patients entering trial and compliance with treatment
Key secondary outcomes	To establish tolerability of trial treatments and procedures

For peer review only - http://bmjopen.bmj.com/site/about/guidelines.xhtml

Figure 1: CIBRAC trial schema

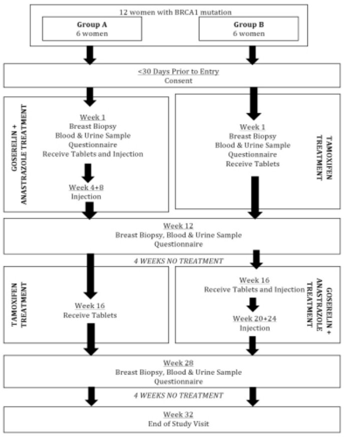

Figure 1

297x209mm (300 x 300 DPI)

List of Abbreviations

AE	Adverse Event
AI	Aromatase Inhibitor
AR	Adverse Reaction
ASNTT	Aseptic Non-touch Technique
BRCA1	Breast Cancer 1 Early Onset Gene
BRCA2	Breast Cancer 2 Early Onset Gene
BRCALink NI	Northern Ireland Support Group for BRCA1 & BRCA2 mutation carriers
CCRCB	Centre for Cancer Research and Cell Biology
CI	Chief Investigator
CIBRAC	Chemoprevention in BRCA1 mutation Carriers Study
CRF	Case Report Forms
DNA	Deoxyribonucleic Acid
DSB	Double Strand Break
EDTA	Ethylenediaminetetraacetic acid
EORTC QLQ-C30	European Organisation for Research and Treatment of Cancer Questionnaire to Quality of Life of Cancer patients
EORTC QLQ-BR23	European Organisation for Research and Treatment of Cancer Questionnaire module to assess quality of life of breast cancer patients and supplement EORTC QLQ-C30
ER	Oestrogen Receptor
FFPE	Formalin-fixed paraffin-embedded
HSC REC B	Health and Social Care Research Ethics Committee B
IBIS-II	International Breast Cancer Interventional Study - Prevention
IMP	Investigational Medicinal Product
Ki67	Antigen KI-67, marker of cellular proliferation
LHRHa	Lutenizing Hormone Releasing Hormone Agonist
NICE	National Institute for Clinical Excellence
ORECNI	Office for Research Ethics Committees Northern Ireland
Participant	An individual who has given written informed consent and is participating in trial related activities
Patient	A patient under care who may be eligible for the trial but has not yet consented to participate in any trial related activities
PIS	Patient Information Sheet
PR	Progesterone Receptor
QOL	Quality of Life
RANK	Receptor Activator of Nuclear Factor κ B
RANKL	Ligand of Receptor Activator of Nuclear Factor κ B
REC	Research Ethics Committee
RNAse	Ribonuclease
SAE	Serious Adverse Event
SAR	Serious Adverse Reaction

SERM	Selective Oestrogen Receptor Modulator
SOP	Standard Operating Procedure
TMG	Trial Management Group
TNF	Tumour Necrosis Factor
UPLC-MS/MS	Ultra Performance Liquid Chromatography Tandem Mass Spectrometry
2-OHE_2	2-hydroxyestradiol
4-OHE_2	4-hydroxyestradiol
53BP1	P53 Binding Protein 1
ϒH2AX	Phosphorylated Histone A2 Variant X

For peer review only

For peer review only

Reporting checklist for protocol of a clinical trial.

Based on the SPIRIT guidelines.

Instructions to authors

Complete this checklist by entering the page numbers from your manuscript where readers will find each of the items listed below.

Your article may not currently address all the items on the checklist. Please modify your text to include the missing information. If you are certain that an item does not apply, please write "n/a" and provide a short explanation.

Upload your completed checklist as an extra file when you submit to a journal.

In your methods section, say that you used the SPIRIT reporting guidelines, and cite them as:

Chan A-W, Tetzlaff JM, Altman DG, Laupacis A, Gøtzsche PC, Krleža-Jerić K, Hróbjartsson A, Mann H, Dickersin K, Berlin J, Doré C, Parulekar W, Summerskill W, Groves T, Schulz K, Sox H, Rockhold FW, Rennie D, Moher D. SPIRIT 2013 Statement: Defining standard protocol items for clinical trials. Ann Intern Med. 2013;158(3):200-207

		Reporting Item	Page Number
Title	#1	Descriptive title identifying the study design, population, interventions, and, if applicable, trial acronym	1
Trial registration	#2a	Trial identifier and registry name. If not yet registered, name of intended registry	1
Trial registration: data set	#2b	All items from the World Health Organization Trial Registration Data Set	21
Protocol version	#3	Date and version identifier	1
Funding	#4	Sources and types of financial, material, and other support	14
Roles and responsibilities: contributorship	#5a	Names, affiliations, and roles of protocol contributors	1,14
Roles and responsibilities:	#5b	Name and contact information for the trial sponsor	14

For peer review only

sponsor contact information			
Roles and responsibilities: sponsor and funder	#5c	Role of study sponsor and funders, if any, in study design; collection, management, analysis, and interpretation of data; writing of the report; and the decision to submit the report for publication, including whether they will have ultimate authority over any of these activities	14
Roles and responsibilities: committees	#5d	Composition, roles, and responsibilities of the coordinating centre, steering committee, endpoint adjudication committee, data management team, and other individuals or groups overseeing the trial, if applicable (see Item 21a for data monitoring committee)	10
Background and rationale	#6a	Description of research question and justification for undertaking the trial, including summary of relevant studies (published and unpublished) examining benefits and harms for each intervention	4-5
Background and rationale: choice of comparators	#6b	Explanation for choice of comparators	4-5
Objectives	#7	Specific objectives or hypotheses	17
Trial design	#8	Description of trial design including type of trial (eg, parallel group, crossover, factorial, single group), allocation ratio, and framework (eg, superiority, equivalence, non-inferiority, exploratory)	6, and Figure 1.
Study setting	#9	Description of study settings (eg, community clinic, academic hospital) and list of countries where data will be collected. Reference to where list of study sites can be obtained	6
Eligibility criteria	#10	Inclusion and exclusion criteria for participants. If applicable, eligibility criteria for study centres and individuals who will perform the interventions (eg, surgeons, psychotherapists)	17
Interventions: description	#11a	Interventions for each group with sufficient detail to allow replication, including how and when they will be administered	6

For peer review only

Interventions: modifications	#11b	Criteria for discontinuing or modifying allocated interventions for a given trial participant (eg, drug dose change in response to harms, participant request, or improving / worsening disease)	6,7
Interventions: adherance	#11c	Strategies to improve adherence to intervention protocols, and any procedures for monitoring adherence (eg, drug tablet return; laboratory tests)	7
Interventions: concomitant care	#11d	Relevant concomitant care and interventions that are permitted or prohibited during the trial	7
Outcomes	#12	Primary, secondary, and other outcomes, including the specific measurement variable (eg, systolic blood pressure), analysis metric (eg, change from baseline, final value, time to event), method of aggregation (eg, median, proportion), and time point for each outcome. Explanation of the clinical relevance of chosen efficacy and harm outcomes is strongly recommended	17
Participant timeline	#13	Time schedule of enrolment, interventions (including any run-ins and washouts), assessments, and visits for participants. A schematic diagram is highly recommended (see Figure)	6, and Figure 1
Sample size	#14	Estimated number of participants needed to achieve study objectives and how it was determined, including clinical and statistical assumptions supporting any sample size calculations	9
Recruitment	#15	Strategies for achieving adequate participant enrolment to reach target sample size	6
Allocation: sequence generation	#16a	Method of generating the allocation sequence (eg, computer-generated random numbers), and list of any factors for stratification. To reduce predictability of a random sequence, details of any planned restriction (eg, blocking) should be provided in a separate document that is unavailable to those who enrol participants or assign interventions	6
Allocation concealment	#16b	Mechanism of implementing the allocation sequence (eg, central telephone; sequentially numbered, opaque, sealed	See note 1

For peer review only

mechanism		envelopes), describing any steps to conceal the sequence until interventions are assigned	
Allocation: implementation	#16c	Who will generate the allocation sequence, who will enrol participants, and who will assign participants to interventions	6
Blinding (masking)	#17a	Who will be blinded after assignment to interventions (eg, trial participants, care providers, outcome assessors, data analysts), and how	See note 2
Blinding (masking): emergency unblinding	#17b	If blinded, circumstances under which unblinding is permissible, and procedure for revealing a participant's allocated intervention during the trial	N/A - non-blinded study
Data collection plan	#18a	Plans for assessment and collection of outcome, baseline, and other trial data, including any related processes to promote data quality (eg, duplicate measurements, training of assessors) and a description of study instruments (eg, questionnaires, laboratory tests) along with their reliability and validity, if known. Reference to where data collection forms can be found, if not in the protocol	10
Data collection plan: retention	#18b	Plans to promote participant retention and complete follow-up, including list of any outcome data to be collected for participants who discontinue or deviate from intervention protocols	See note 3
Data management	#19	Plans for data entry, coding, security, and storage, including any related processes to promote data quality (eg, double data entry; range checks for data values). Reference to where details of data management procedures can be found, if not in the protocol	See note 4
Statistics: outcomes	#20a	Statistical methods for analysing primary and secondary outcomes. Reference to where other details of the statistical analysis plan can be found, if not in the protocol	9
Statistics: additional analyses	#20b	Methods for any additional analyses (eg, subgroup and adjusted analyses)	9
Statistics: analysis population and missing data	#20c	Definition of analysis population relating to protocol non-adherence (eg, as randomised analysis), and any statistical methods to handle missing data (eg, multiple	9

For peer review only

		imputation)	
Data monitoring: formal committee	#21a	Composition of data monitoring committee (DMC); summary of its role and reporting structure; statement of whether it is independent from the sponsor and competing interests; and reference to where further details about its charter can be found, if not in the protocol. Alternatively, an explanation of why a DMC is not needed	10
Data monitoring: interim analysis	#21b	Description of any interim analyses and stopping guidelines, including who will have access to these interim results and make the final decision to terminate the trial	10
Harms	#22	Plans for collecting, assessing, reporting, and managing solicited and spontaneously reported adverse events and other unintended effects of trial interventions or trial conduct	7
Auditing	#23	Frequency and procedures for auditing trial conduct, if any, and whether the process will be independent from investigators and the sponsor	10
Research ethics approval	#24	Plans for seeking research ethics committee / institutional review board (REC / IRB) approval	11
Protocol amendments	#25	Plans for communicating important protocol modifications (eg, changes to eligibility criteria, outcomes, analyses) to relevant parties (eg, investigators, REC / IRBs, trial participants, trial registries, journals, regulators)	19
Consent or assent	#26a	Who will obtain informed consent or assent from potential trial participants or authorised surrogates, and how (see Item 32)	6/7
Consent or assent: ancillary studies	#26b	Additional consent provisions for collection and use of participant data and biological specimens in ancillary studies, if applicable	N/A
Confidentiality	#27	How personal information about potential and enrolled participants will be collected, shared, and maintained in order to protect confidentiality before, during, and after the trial	See note 5
Declaration of	#28	Financial and other competing interests for principal	14

For peer review only

interests		investigators for the overall trial and each study site	
Data access	#29	Statement of who will have access to the final trial dataset, and disclosure of contractual agreements that limit such access for investigators	See note 6
Ancillary and post trial care	#30	Provisions, if any, for ancillary and post-trial care, and for compensation to those who suffer harm from trial participation	N/A
Dissemination policy: trial results	#31a	Plans for investigators and sponsor to communicate trial results to participants, healthcare professionals, the public, and other relevant groups (eg, via publication, reporting in results databases, or other data sharing arrangements), including any publication restrictions	11
Dissemination policy: authorship	#31b	Authorship eligibility guidelines and any intended use of professional writers	11
Dissemination policy: reproducible research	#31c	Plans, if any, for granting public access to the full protocol, participant-level dataset, and statistical code	11
Informed consent materials	#32	Model consent form and other related documentation given to participants and authorised surrogates	See note 7
Biological specimens	#33	Plans for collection, laboratory evaluation, and storage of biological specimens for genetic or molecular analysis in the current trial and for future use in ancillary studies, if applicable	See note 8

For peer review only

Author notes

1. N/A (non-blinded study)
2. N/A (non-blinded study)
3. Outlined in CIBRAC Data Management Plan
4. Outlined in CIBRAC Data Management Plan
5. Outlined in CIBRAC Data Management Plan
6. Outlined in CIBRAC Data Management Plan
7. N/A - in Trial Master File

8. N/A - in CIBRAC Laboratory Manual

The SPIRIT checklist is distributed under the terms of the Creative Commons Attribution License CC-BY-ND 3.0. This checklist was completed on 20. March 2018 using http://www.goodreports.org/, a tool made by the EQUATOR Network in collaboration with Penelope.ai

For peer review only
